# Multi‐Habitat Landscape Promotes Microbial Diversity: Insights from the Traditional Agricultural Heritage and the Global Trend

**DOI:** 10.1002/advs.202506402

**Published:** 2025-09-23

**Authors:** Jintao He, Jian Xiao, Xiaoqiang Shen, Kankan Zhao, Xiaoyu Lei, Huarui Zhang, Chao Sun, Huijie Lu, Yongqi Shao

**Affiliations:** ^1^ Max Planck Partner Group Institute of Sericulture and Apiculture Faculty of Agriculture Life and Environmental Sciences Zhejiang University Hangzhou 310058 China; ^2^ Institute of Soil and Water Resources and Environmental Science College of Environmental and Resource Sciences Zhejiang University Hangzhou 310058 China; ^3^ Analysis Center of Agrobiology and Environmental Sciences Zhejiang University Hangzhou 310058 China; ^4^ Key Laboratory of Environmental Remediation and Ecological Health Ministry of Education College of Environmental and Resource Sciences Zhejiang University Hangzhou 310058 China; ^5^ Key Laboratory of Water Pollution Control and Environmental Safety Zhejiang 310058 China; ^6^ Key Laboratory of Silkworm and Bee Resource Utilization and Innovation of Zhejiang Province Hangzhou 310058 China; ^7^ Key Laboratory for Molecular Animal Nutrition Ministry of Education Hangzhou 310058 China

**Keywords:** dispersal assembly, ecosystem microbiome, habitat diversity and heterogeneity, microbial diversity, microdiversity, Mulberry‐dyke and Fish‐pond, traditional agricultural system

## Abstract

Ecosystems are interconnected networks of diverse habitat types, rather than isolated patches. However, the role of the multi‐habitat landscape in influencing microbial diversity remains poorly understood. This study investigates bacterial and fungal communities within a 2500‐year agricultural heritage system, the Mulberry‐dyke and Fish‐pond (MF), which integrates various terrestrial and aquatic habitats. Using amplicon sequencing, metagenomics, metatranscriptomics, and genomic analyses, these findings reveal a significant proportion of unclassified microbial taxa, underscoring the importance of MF systems as an untapped reservoir of microbial genetic resources. Moreover, single‐nucleotide‐level analyses demonstrate that a multi‐habitat landscape enhances microbial diversity through ecosystem‐wide assembly, facilitated by cross‐habitat microbial dispersal. Taxa found across multiple habitats exhibit convergence in microdiversity and adaptive genetic traits, indicating both ecological and functional mechanisms underlying their adaptability. A global analysis of public microbiome datasets furthermore confirms that regions with higher habitat heterogeneity support significantly higher taxonomic and functional diversity of microbiomes. Overall, this study sheds new light on the overlooked microbial diversity in traditional agricultural heritages and emphasizes the value of ancestral ecological wisdom underlying multi‐habitat integration for ecosystem management. These insights offer valuable guidance for developing sustainable agricultural strategies, enhancing microbial diversity, and reinforcing ecosystem resilience in the face of global change.

## Graphical abstract

The 2500‐year‐old Mulberry‐dyke and Fish‐pond agricultural heritage system reveals how interconnected habitats foster microbial diversity. Multi‐habitat landscapes enhance microbial diversity through cross‐habitat dispersal and adaptive trait convergence. Globally, regions with diverse habitats support a greater diversity of soil microbiomes. The ancestral wisdom from agricultural heritage unveils the value of habitat diversity for sustainable agriculture and ecosystem resilience.

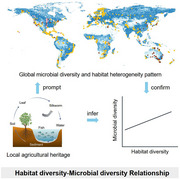

## 1 Introduction

Ecosystem functionality and stability are intricately linked to the biodiversity of macroorganisms and microorganisms in diverse habitats.^[^ ^1^, ^2^, ^3^ ^]^ Recently, Hackett et al. highlighted that multi‐habitat landscapes exhibit greater diversity and stability with improved function,^[^ ^4^ ^]^ emphasizing ecosystems as interconnected networks rather than isolated patches. While microorganisms play pivotal roles in ecosystem services, the effects of multi‐habitat landscapes on microbial diversity remain poorly understood. Research in microbial ecology increasingly challenges the Baas‐Becking hypothesis^[^ ^5^ ^]^—“Everything is everywhere, but the environment selects”, which the niche‐assembly theory underlies (namely, niches limit the maximum number of coexisting species). Evidence suggests that microbial communities may not be solely shaped by local environmental conditions but are also affected by cross‐habitat dispersal processes,^[^ ^6^, ^7^ ^]^ with certain microbes exhibiting the ability to maintain viability and functionality in distinct habitat types.^[^ ^8^, ^9^ ^]^ These cross‐habitat microbes could play important roles in shaping local community diversity, function, and even evolutionary processes by facilitating gene turnover.^[^ ^10^, ^11^ ^]^ For example, cross‐habitat dispersal can enhance local biodiversity by increasing species richness;^[^ ^12^ ^]^ immigrant individuals may function similarly to local species (redundancy) or occupy empty ecological niches (complementarity), thereby enhancing functionality and stabilizing functional variability under environmental perturbations.^[^ ^4^ ^]^ However, microbial studies have predominantly focused on single habitats, often overlooking the broader impacts of multi‐habitat landscapes. This gap in understanding hinders our ability to predict and manage microbiome functionality and resilience at the ecosystem scale.^[^ ^13^ ^]^

Traditional agricultural systems (TASs) present a unique opportunity to study the multi‐habitat landscapes of microbial communities. For centuries, these systems have employed ecological strategies that significantly contribute to global food production and biodiversity conservation.^[^ ^14^, ^15^ ^]^ Unlike modern agricultural systems, which are often simplified and intensified, TASs typically integrate multiple habitats to optimize resource utilization and promote species coexistence.^[^ ^16^ ^]^ For instance, the rice‐fish coculture system illustrates how habitat complementarity supports genetic diversity and improves resilience to environmental stress.^[^ ^17^, ^18^, ^19^ ^]^ Notably, the abandonment of TASs could potentially elevate species extinction rates by more than two orders of magnitude.^[^ ^16^ ^]^ Particularly, the microbial diversity within traditional agricultural heritages remains underexplored.^[^ ^20^, ^21^, ^22^ ^]^

**Figure 1:**
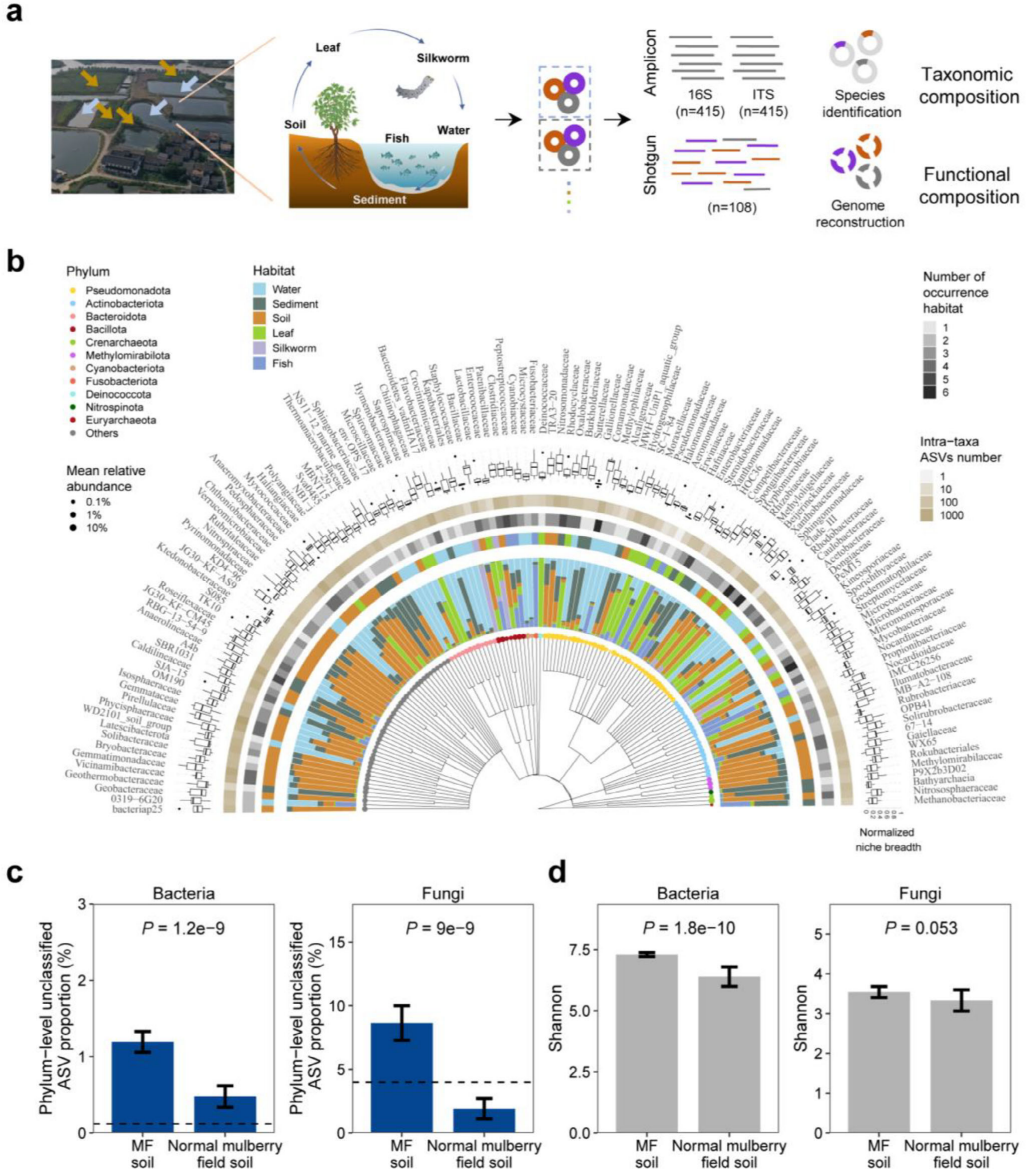
Unexplored microbial diversity and novelty within the MF system. a) The left panel shows representative Mulberry‐dyke and Fish‐pond (MF) systems (brown and light blue arrows indicate mulberry dyke and fish pond, respectively). The diagram describes the traditional strategy of integrating multiple habitats: mulberry leaves feed silkworms, whose waste nourishes pond fish; in turn, fish excrete waste that enriches the pond sediment, which is later utilized as organic fertilizer for mulberry trees, thereby promoting matter cycling and energy flow. The right panel indicates the workflow of this study. Microbiomes from six major habitats (water, sediment, soil, mulberry leaf, silkworm gut, and fish gut) within MF systems were examined using amplicon sequencing of conserved phylogenetic marker genes for bacteria and fungi, along with shotgun sequencing to determine the functional composition. After quality control and sample pairing, a total of 415 pairs of bacterial and fungal amplicon data were obtained from the six habitats, including water, sediment, soil, mulberry leaf, silkworm gut, and fish gut (*n* = 99, 52, 157, 77, 12, and 18, respectively). b) The taxonomic cladogram shows major families observed in the 16S rRNA amplicon data. The color of each tipping point represents the phylum. The inner bar plots indicate the relative proportion of each family among habitats. For example, a bar filled with 50% light blue and 50% blue indicates that the phylum occurs only in water and fish habitats with equal relative abundance. Three rings represent the enriched habitat, the number of occurrence habitats, and the number of ASVs of each family. External boxplots indicate the normalized niche breadth of the family within each habitat. c,d) MF exhibits greater novelty and diversity of soil microbiota (*n* = 157) compared to normal mulberry field soil (*n* = 28) and global soil (*n* = 235) (rarefied to 10 000). Different from MF, normal mulberry fields employ conventional mulberry planting practices without integrated fishponds (low habitat diversity). Dashed lines represent the documented average proportion of phylum‐level unknown taxa in global soil. Microbial novelty is indicated by the proportion of phylum‐level unknown ASVs within communities. The same version of taxonomic database (Greengenes v13.8 and UNITE v8.0 for bacteria and fungi, respectively) was used for the annotation and comparison between datasets. Data are presented as mean ± standard error of the mean. *P* values are calculated using the Wilcoxon rank‐sum test.

As a Globally Important Agricultural Heritage System (GIAHS), the Mulberry‐dyke and Fish‐pond (MF),^[^ ^23^ ^]^ an ancient multi‐habitat agricultural system in Huzhou, China, serves as a prime example of a TAS that thrived for 2500 years. This ancient multi‐habitat system integrates diverse aquatic and terrestrial environments, as well as host and non‐host habitats (**Figure** **1**a). It produces a variety of products, including mulberry leaves, fruits, fish, silk, and nutrient‐rich pupae. Primarily found in China's Taihu Lake Basin (notably Huzhou City) and the Pearl River Delta, MF systems are crucial for both economic sustainability and ecological health, as they support biodiversity, sericulture, and aquaculture. In this study, we analyzed the dynamics of bacterial and fungal communities across six major habitats within the MF system: Water, Sediment, Soil, Leaf (mulberry phyllosphere), Silkworm (invertebrate gut), and Fish (vertebrate gut). Using amplicon sequencing (*n* = 830), metagenomics (*n* = 108), metatranscriptomics (*n* = 24), and comparative genomics (*n* = 1182), we aim to: 1) investigate the unexplored microbial diversity within MF systems; 2) examine how multi‐habitat landscapes influence microbial diversity; 3) identify the adaptive mechanisms mediating microbial cross‐habitat distribution; and 4) assess the global‐scale pattern using public datasets. We hypothesize that multi‐habitat landscapes can promote microbial diversity through dispersal processes even among distinct habitat types. By revealing how a multi‐habitat agricultural heritage system sustains microbial diversity, this study underscores the significant importance of ancestral agricultural legacy and practices in microbial ecology and the management of agroecosystems.

## 2 Results

### 2.1 Novel Microbial Diversity and Inter‐Habitat Connectivity Within MF Systems

Amplicon sequencing and rigorous data processing generated 46811 bacterial amplicon sequence variants (ASVs) and 9445 fungal ASVs (Figure 1a; Figure S1, Supporting Information). These ASVs spanned 743 bacterial families (75 phyla) and 409 fungal families (16 phyla) (Figure 1b). Notably, taxonomic assignment showed that on average, 1.19% (bacteria) and 8.64% (fungi) of ASVs within communities could not be assigned even at the phylum level. Taxa classified as bacteria/fungi but not assigned at the phylum level may represent potential new phyla.^[^ ^24^ ^]^ We found that the MF soils exhibited significantly more phylum‐level unclassified ASVs compared to normal mulberry field soils, which were collected from seven major cultivation provinces in China and processed following identical protocols (*P* < 0.001, Figure 1c). This finding is consistently observed when compared to reported global averages (0.12% for bacteria and 3.98% for fungi)^[^ ^24^ ^]^ (*P* < 0.001, one‐sample t‐test). Concordantly, metagenome‐assembled genomes (MAGs) from MF soil exhibited greater novelty compared to MAGs from the global soil genome catalogue^[^ ^25^ ^]^ (*P* < 0.001, Welch's t‐test, Figure S2, Supporting Information), as evidenced by lower values of maximal average nucleotide identity to GTDB reference genomes.

To test our hypothesis regarding the role of multi‐habitat landscapes, we compared the soil microbiomes of MF systems with those of normal mulberry fields that lack fishpond integration (low habitat diversity). The results showed that MF soils exhibited higher microbial diversity than those of normal mulberry field soils (Figure 1d), suggesting the role of multi‐habitat landscapes in enhancing microbial diversity. To investigate potential inter‐habitat linkages, we examined the distribution and adaptability of microbial families across diverse habitats using two metrics: the average relative abundance and Levin's niche breadth index (Figure 1b). The niche breadth index, which infers the range of environmental conditions in which a microorganism can thrive, is calculated based on the taxon's occurrence across samples.^[^ ^26^ ^]^ Most microbial families exhibited clear habitat preferences, with higher relative abundances in specific habitats (Figure 1b). Families such as Comamonadaceae and Sphingomonadaceae showed broad prevalence and adaptability across all six habitats, as evidenced by their niche breadth indices (Figure S3, Supporting Information).

**Figure 2:**
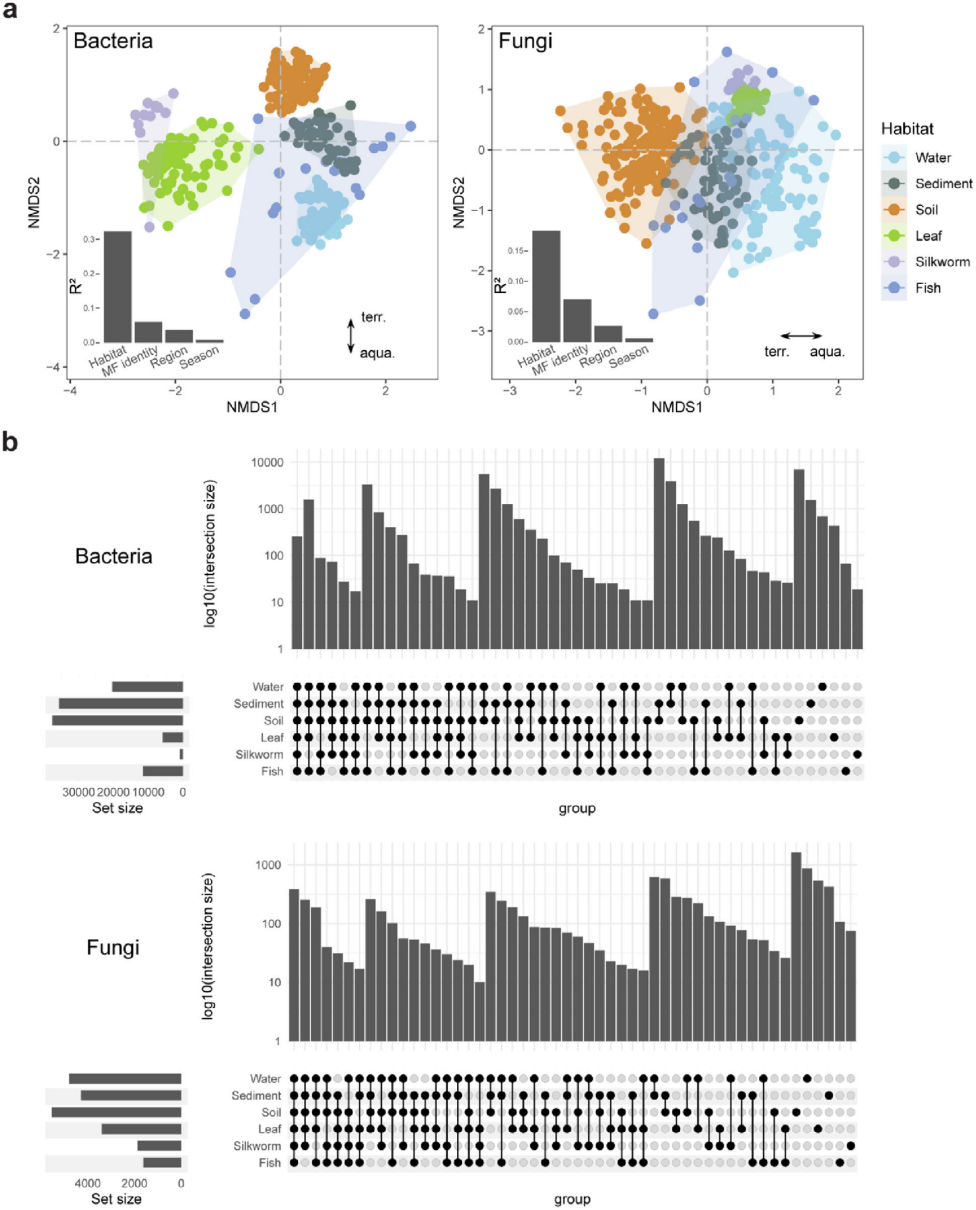
Differentiation and connectivity between habitats. a) Nonmetric multidimensional scaling ordination (NMDS) of bacterial and fungal communities across habitats. The subplots demonstrate the explained variation in bacterial and fungal communities based on factors such as habitat, MF identity, sampling region, and seasonality, using permutational multivariate analysis of variance (PERMANOVA). All factors were statistically significant (*P* < 0.001). Arrows indicate clustering between aquatic and terrestrial habitats. A total of 415 pairs of bacterial and fungal amplicon data from six habitats were analyzed: *n* = 99, 52, 157, 77, 12, and 18, respectively (water, sediment, soil, mulberry leaf, silkworm gut, and fish gut). b) Upset plots showing the overlap in bacterial and fungal ASVs between habitats. The left panel indicates the number of ASVs in each habitat. The right panel shows an intersection matrix displaying the group in which habitats were detected. Bar plot displays the log‐transformed number of ASVs in the intersection.

Beta diversity analysis at the ASV level revealed that habitat type significantly influenced both bacterial and fungal communities (R^2^ = 0.32 and 0.18, respectively; *P* < 0.001). This was followed by MF identity, representing the sampling sites (21 different MF) (R^2^ = 0.06 and 0.07, respectively; *P* < 0.001) (**Figure** **2**a). The null model‐based analysis suggested that selection and dispersal‐related processes play roles in shaping microbiomes across habitats (Figure S4, Supporting Information). To further assess inter‐habitat connectivity, we counted the number of shared bacterial and fungal ASVs across habitats (Figure 2b). While each habitat contained unique ASVs, a remarkable degree of bacterial and fungal ASVs were shared across habitats, even between habitats that were not directly linked by the cycling agricultural practices of MF. Among the intersections of five habitats, the bacterial intersection excluding silkworm gut and the fungal intersection excluding fish gut exhibited a higher number of shared ASVs. Notably, a total of 257 (bacteria) and 384 (fungi) ASVs were shared across all habitats, highlighting inter‐habitat connectivity.

Collectively, these results unveil microbial diversity within MF systems and highlight the potential to uncover previously untapped microbial genetic resources from traditional agricultural heritage systems. Additionally, the patterns observed in this unique system support the hypothesis that multi‐habitat landscapes influence microbial diversity. We then used the MF system as a research model to illustrate microbial inter‐habitat connectivity.

### 2.2 Multi‐Habitat Landscape Promotes Microbial Diversity Within MF Systems

Recent ecological studies indicate that many species within a community do not co‐exist stably but instead transiently co‐occur due to dispersal from other habitats.^[^ ^12^, ^27^, ^28^ ^]^ These transient taxa play an important role in sustaining community diversity. For example, microorganisms in the silkworm gut are often sourced from mulberry leaf diets (nonnative) and are considered transient due to distinct environmental conditions, such as the extremely alkaline pH of the silkworm gut.^[^ ^29^, ^30^, ^31^ ^]^ Such transient or nonnative taxa are expected to exhibit lower abundance and adaptability in nonnative habitats.^[^ ^26^, ^27^, ^32^ ^]^ In contrast, taxa that successfully establish in a habitat tend to exhibit higher abundance or wider niche breadth, indicating enhanced adaptive capacity.

**Figure 3:**
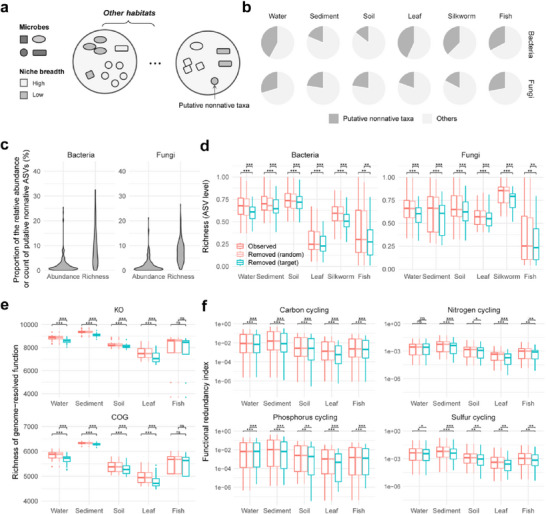
Inter‐habitat connectivity within MF systems contributes to microbial diversity through putative nonnative taxa. a) Schematic representation of the identification of potential nonnative species. For each pair of habitats and species, species were categorized as putative nonnative only when they exhibited: a lower niche breadth index compared to indices calculated from other species within the habitat; a lower niche breadth index than indices calculated from other habitats; and a lower relative abundance than in other habitats. b) Proportion of putative nonnative ASVs within the metacommunity (the set of all communities) across habitats. c) Relative abundance (Abundance) and proportion (Richness) of putative nonnative taxa within communities (*n* = 415). d) Taxonomic richness (normalized by the maximum richness values in each habitat) in observed and simulated communities across habitats, including water, sediment, soil, mulberry leaf, silkworm gut, and fish gut (amplicon data; *n* = 99, 52, 157, 77, 12, and 18, respectively). Simulated communities were generated by two methods: 1) targeted removal of putative nonnative taxa, marked as Removed (target), and 2) random removal of an equivalent number of taxa, marked as Removed (random). e,f) Functional richness (e) and functional redundancy (f) in observed and simulated communities, using MAGs (*n* = 1182) and metagenomes (*n* = 108). Metagenome sample number: 28, 27, 28, 16, and 9 from water, sediment, soil, mulberry leaf, and fish gut, respectively. Functional richness was calculated based on KEGG Orthology (KO) and Clusters of Orthologous Genes (COG) annotations from MAGs. Functional redundancy indices of four biogeochemical processes (carbon, nitrogen, phosphorus, and sulfur) were calculated based on KO annotation of MAGs. Functional redundancy index predicts resilience to functional loss during environmental disturbances, with higher values indicating greater redundancy. Boxplots show the median (line), 25th and 75th percentiles (box), and 1.5 × the interquartile range (whiskers). Outliers are represented by dots. Differences between groups in each habitat were evaluated using the paired t‐test and adjusted using the false discovery rate. (*P ≤ 0.05, **P ≤ 0.01, ***P ≤ 0.001, and ns = *P* > 0.05).

To classify putative nonnative taxa, we employed a dual‐criterion approach based on relative abundance and niche breadth index (**Figure** **3**a, details provided in Experimental Section). This approach allowed us to identify taxa that act as indicators of inter‐habitat connectivity. On average, 31.4% of bacterial ASVs and 23.3% of fungal ASVs within each habitat were classified as potentially nonnative (Figure 3b). These putative nonnative taxa showed significantly higher contributions to bacterial richness in host‐associated communities (averaging 39.0% and 27.2% for host and non‐host communities, respectively) compared to fungal richness (averaging 22.3% and 25.1% for host and non‐host communities, respectively) (Figure 3b), suggesting their varying roles in different habitat types. To verify the origins of putative nonnative taxa, we utilized metagenomic datasets and employed single nucleotide variant‐based source tracking analysis.^[^ ^33^ ^]^ Compared to ASV‐level analysis, this approach offers greater precision in detecting potential microbial movement across habitats. We analyzed 108 metagenomes and 1182 nonredundant MAGs from the genome catalog of the MF system. This analysis revealed that genome variants of putative nonnative taxa in each habitat were strongly linked to microbial populations in other habitats (Figure S5, Supporting Information). For example, taxa identified as nonnative in fish guts exhibited genetic signatures predominantly traceable to metagenomes from other habitats (90.0%). These findings confirmed that these nonnative taxa were not indigenous to their current habitats but were likely introduced via cross‐habitat microbial dispersal. To address the possibility that these taxa might merely represent relic DNA—DNA from extracellular or dead cells that could falsely suggest microbial presence and activity^[^ ^34^ ^]^—we conducted a metatranscriptomic analysis (Figure S6, Supporting Information). The results demonstrated that a significant proportion of the putative nonnative taxa were transcriptionally active, confirming their functional activity and ecological relevance, and supporting the critical roles of multi‐habitat landscapes in shaping ecosystem microbiomes.

The structure and function of local communities can be influenced by the dispersal of organisms between different habitats.^[^ ^4^, ^35^ ^]^ To quantify the contributions of putative nonnative taxa, we compared observed microbial communities with simulated communities that excluded these taxa. Despite representing only 2.4% (bacteria) and 2.2% (fungi) of relative abundance, these taxa significantly enhanced community richness: contributing 9.1% (bacteria) and 7.8% (fungi) to overall taxonomic diversity (Figure 3c). Further analysis demonstrated that communities with targeted removal of nonnative taxa exhibited significantly lower taxonomic richness than both observed communities and simulated scenarios with random taxon removal (Figure 3d). Nonnative taxa may also contribute to functional complementarity and redundancy. To further assess their contributions, we examined community functional richness and the functional redundancy index using MAG‐based functional profiles (Figure 3e,f). The silkworm gut habitat was excluded from the analysis due to technical issues in obtaining microbial reads from significant host contamination. We found that functional richness was significantly lower in simulated communities lacking putative nonnative taxa compared to observed communities as well as simulations with random taxon removal (Figure 3e). Additionally, functional redundancy indices, measured via phylogenetic distribution of community members harboring key biogeochemical functions, were also significantly reduced in communities without putative nonnative taxa (Figure 3f).

Collectively, these findings highlight the essential roles of inter‐habitat connectivity in promoting the diversity and function of microbial communities. The disproportionate contributions of nonnative taxa to both taxonomic and functional diversity emphasize the importance of multi‐habitat landscapes for maintaining ecosystem functionality.

### 2.3 Ecological and Functional Convergence Reveals Adaptive Mechanisms of Cross‐Habitat Taxa

**Figure 4:**
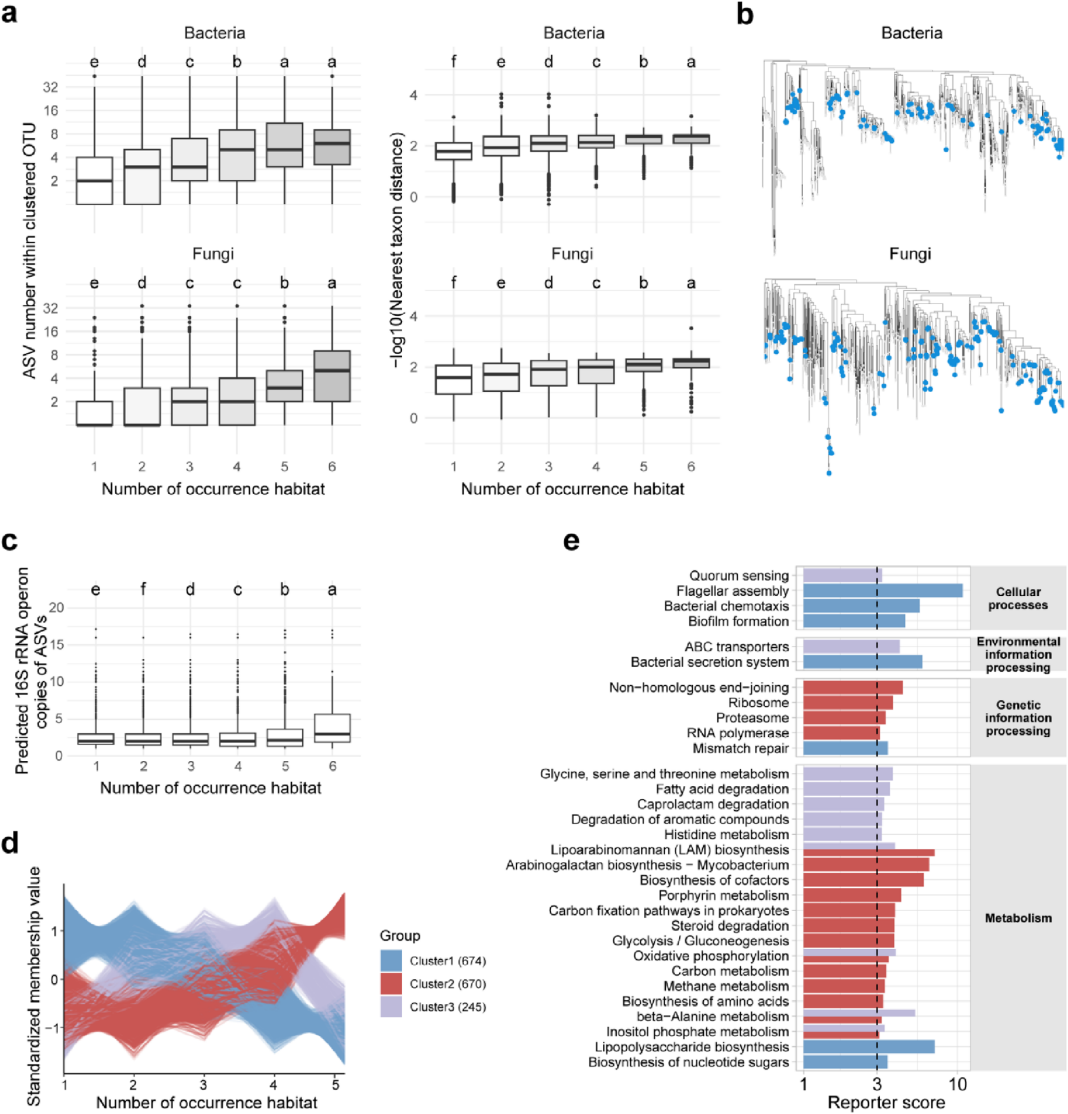
Convergent ecological patterns and functional capacity of cross‐habitat taxa. a) Higher microdiversity of taxa associated with multiple habitats. The left panel illustrates the number of ASVs within bacterial (*n* = 6048, 13799, 8104, 3410, 1188, and 174, respectively) and fungal (*n* = 1660, 1689, 1223, 629, 409, and 283, respectively) OTUs that occur in different numbers of habitats from 1 to 6. The right panel shows the nearest taxon closeness (represented by the minus log‐transformed nearest taxon distance) of bacterial (*n* = 9743, 18772, 11094, 5139, 1806, and 257, respectively) and fungal (*n* = 3652, 2566, 1475, 816, 552, and 384, respectively) ASVs that occur in different numbers of habitats. Higher values indicate greater ecological fine‐scale diversification. Colors represent the number of occurrence habitats. b) The trees show widespread phylogenetic distributions of representative ASVs (in blue) that were detected across all six habitats. c) Predicted 16S rRNA operon copies of bacterial ASVs that occur in different numbers of habitats. d) C‐means clustering of KO gene profiles across MAGs that occur in different numbers of habitats. The y‐axis represents standardized membership values. Genes with membership scores greater than 0.7 are displayed. e) Functional enrichment analysis based on generalized Reporter Score‐based analysis (Reporter score > 3). The x‐axis is log‐transformed. Boxplots show the median (line), 25th and 75th percentiles (box), and 1.5 × the interquartile range (whiskers). Outliers are represented by dots. Letters in each boxplot indicate significance (Figure 4a,c). Groups that do not share a letter are significantly different (false discovery rate‐adjusted *P* < 0.05, pair‐wise t‐test).

A multi‐habitat landscape could promote microbial diversity in each habitat through dispersal and also favor microorganisms that can adapt to multiple habitats. Recent studies have reported that microbial taxa with broad spatial niches are often microdiverse at the ecological level (e.g., ASVs within operational taxonomic units (OTUs)), contributing to their high prevalence.^[^ ^36^ ^]^ Therefore, we examined the microdiversity of taxa with broad spatial niches at the ecosystem scale (those present in multiple habitat types) within the MF system. By comparing the number of ASVs within OTUs associated with different numbers of habitats (**Figure** **4**a), we found that cross‐habitat OTUs exhibited a greater number of intra‐OTU ASVs. At the ASV level, cross‐habitat ASVs had more similar closest relatives compared to those restricted to fewer habitat types. In other words, these cross‐habitat taxa consistently exhibited greater ecological fine‐scale diversification, supporting their increased microdiversity. Collectively, these results reveal the ecological convergence in greater microdiversity among cross‐habitat taxa, contributing to their high prevalence across habitats.^[^ ^36^ ^]^

Furthermore, we found that these cross‐habitat ASVs spanned a wide taxonomic and phylogenetic range (Figure 4b; Figure S7, Supporting Information). Consistently, MAGs from families, such as Mycobacteriaceae, Nitrososphaeraceae, and Burkholderiaceae, were observed across all examined habitats, aligning with their known widespread environmental distribution.^[^ ^37^, ^38^, ^39^, ^40^ ^]^ This prevalence raises questions about genetic mechanisms, such as genomic simplification or adaptive gene acquisition, in cross‐habitat taxa. Analysis of 16S rRNA operon copy predictions revealed that cross‐habitat ASVs tended to possess higher counts of rRNA operon copies (Figure 4c). This feature potentially allows them to achieve faster maximal growth rates and respond quickly to environmental resources, characteristics typical of r‐strategists.^[^ ^41^ ^]^ Supporting this, minimal doubling times predicted from codon usage statistics of MAGs^[^ ^42^ ^]^ corroborated their capability for rapid growth (Figure S8, Supporting Information). Furthermore, genomic annotation based on MAGs indicated that genes encoded by MAGs with varying numbers of occurrence habitats clustered into three distinct groups: Cluster 1, which was depleted in cross‐habitat species, and Cluster 2/3, which were enriched in them (Figure 4d). Depleted functional pathways included flagellar assembly, chemotaxis, biofilm formation, secretion systems, and mismatch repair (Figure 4e). In contrast, functions related to genetic information processing, such as non‐homologous end‐joining and ribosome, were enriched, which is consistent with their higher microdiversity and fast growth. Several metabolism‐related pathways, including cofactor synthesis, amino acid synthesis, and carbon metabolism, were also enriched (Figure 4e). Overall, these ecological and functional convergences shed light on the adaptive mechanisms of the cross‐habitat taxa.

### 2.4 Habitat and Microbial Diversity Relationship on a Global Scale

**Figure 5:**
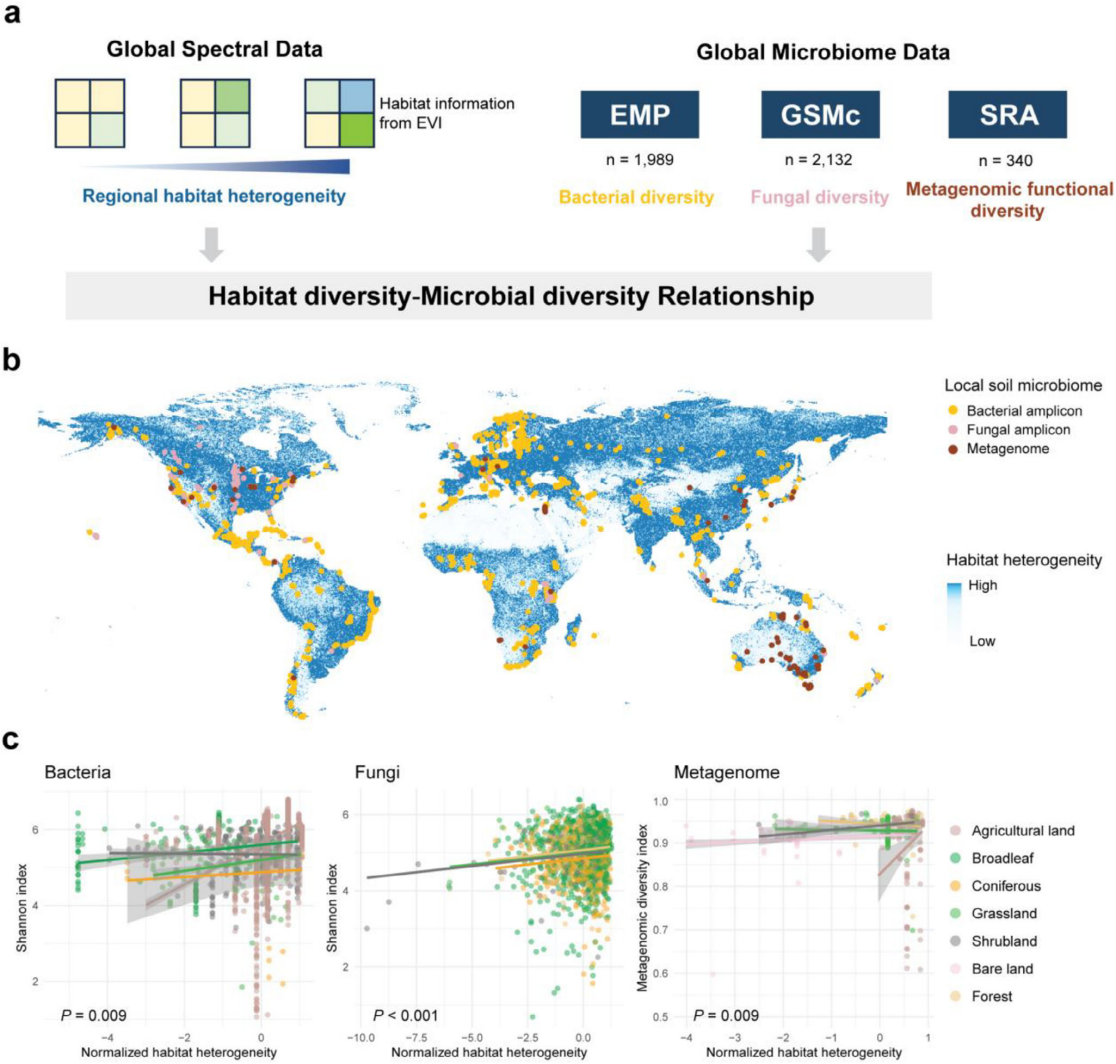
Habitat diversity is positively correlated to microbial taxonomic and functional diversity at the global scale. a) Schematic workflow illustrating the integration of global spectral data and microbiome data. The Enhanced Vegetation Index (EVI), which ranges from −1 to +1, provides valuable habitat information: high scores (above 0.2) indicate vegetation (depicted as green in the left panel's schematic diagram), low scores indicate soil/built areas (light yellow) and water sources (light blue). Habitat diversity is inferred from the heterogeneity of EVI within the region (Global Habitat Heterogeneity metrics) at a 1 km resolution. The right panel displays various global soil microbiome data retrieved from the Earth Microbiome Project (EMP), Global Soil Microbiome Consortium (GSMc), and Sequence Read Archive (SRA) to evaluate local bacterial, fungal, and metagenomic diversity. b) Global habitat heterogeneity and analyzed soil amplicon and metagenomic datasets. The map color demonstrates global patterns of habitat heterogeneity measured by Shannon metrics at a 1 km spatial resolution. Points represent sampling locations for soil bacterial amplicon (*n* = 1989), fungal amplicon (*n* = 2132), and metagenomic (*n* = 340) datasets. c) Positive correlations between habitat heterogeneity and microbial taxonomic/functional diversity metrics. Point colors represent land cover types. Linear mixed models were performed, controlling for study identity, soil type, and climatic factors, and *P* values were shown.

The patterns observed within MF systems reveal that diverse habitats can act as species reservoirs for one another, thereby increasing species pools. Consequently, landscapes with more diverse habitat types tend to support higher microbial community diversity within each habitat. This observation raises a compelling question: can these findings be extrapolated to a global scale? To address this, it is essential to recognize that the concept of habitat diversity, often termed habitat heterogeneity,^[^ ^43^ ^]^ is context‐dependent (Figure S9, Supporting Information). In our cases, the six distinct habitat types within MF systems represent an intuitive form of habitat diversity; however, quantifying such diversity directly in field research is challenging. Remote sensing techniques, combining spectral data with advanced algorithms, are widely employed to assess habitat information. For instance, the enhanced vegetation index (EVI), ranging from −1 to +1, can indicate various habitat types: scores above 0.2 represent vegetation cover, low positive scores denote bare soil or built areas, and negative scores indicate water sources.^[^ ^44^ ^]^ Habitat diversity/heterogeneity can then be inferred from spectral heterogeneity^[^ ^45^, ^46^, ^47^, ^48^ ^]^ based on the textural features of EVI imagery, such as the Global Habitat Heterogeneity index.^[^ ^49^ ^]^ Remote sensing studies have demonstrated that habitat diversity positively influences the diversity of macroorganisms, including insects, birds, reptiles, and mammals.^[^ ^50^, ^51^, ^52^, ^53^ ^]^ However, despite the critical role of microorganisms in ecosystem functionality and services, the relationship between habitat diversity and microbial communities is still unexplored. To bridge this gap, we investigated the relationship between microbial diversity and habitat diversity within their geographic region, using the Global Habitat Heterogeneity index as a proxy for habitat diversity (**Figure** **5**a). Soil microbial communities were analyzed due to their ecological significance and the abundance of available data (Figure 5a). We analyzed 1989 soil bacterial and 2132 fungal communities^[^ ^54^, ^55^ ^]^ in relation to habitat heterogeneity at a 1 km spatial resolution (Figure 5b). Significant positive relationships were observed between habitat heterogeneity and microbial diversity after controlling for study identity, soil type, and climatic factors (*P* = 0.009 and *P* = 6 × 10^−6^ in bacteria and fungi, respectively; linear mixed model) (Figure 5c; Table S1, Supporting Information). Furthermore, we examined the functional diversity of global soil metagenomes (*n* = 340) and found a consistent positive relationship between habitat heterogeneity and functional diversity (*P* = 0.009, linear mixed model). These outcomes indicate that regions with higher habitat diversity tend to support greater taxonomic and functional diversity in local microbial communities. Additional microdiversity analyses confirmed similar trends, reinforcing the association between habitat diversity and microbial diversity (Figure S10, Supporting Information).

## 3 Discussion

In the Anthropocene—an era marked by unprecedented human impact—we are witnessing a crisis in global ecosystem services and resilience, due to reduced habitat complexity and species extinction.^[^ ^56^, ^57^ ^]^ TASs have long harnessed ecological strategies that not only optimize agricultural productivity but also support biodiversity.^[^ ^58^, ^59^ ^]^ Our study provides the first microbiological demonstration of how these ancestral agricultural heritages serve as significant reservoirs of microbial diversity, containing novel microbial genetic resources. Notably, MF soil harbors a greater proportion of undescribed taxa compared to global soil averages, suggesting the uncharted microbial “dark matter” within underexplored MF systems. The accurate assessment of novel taxa can be influenced by factors such as sequencing platforms, primer selection biases, and database limitations. For instance, primer choice may skew results by preferentially excluding or underrepresenting certain phylogenetic groups.^[^ ^60^ ^]^ Future incorporation of complementary approaches like culturomics may help resolve these unclassified lineages and enable more robust characterization of microbial genetic novelty within these systems. Given the steep decline of traditional agriculture and the threats posed by climate change, it is increasingly urgent to prioritize the preservation and detailed characterization of the unique microbial genetic resources. Efforts to document microbial resources in vulnerable ecosystems are already underway; for example, metagenomic sequencing has been used to reconstruct microbial genomes and compile genome catalogs in melting glaciers, which are threatened by global warming.^[^ ^61^ ^]^ In the future, we aim to build gene and genome catalogs from extensive metagenomic data in MF systems and other important TASs, ensuring that these invaluable genetic resources are archived for conservation, biotechnological applications, and microbial ecology research.

A central goal of microbial ecology is to clarify the processes and mechanisms driving microbial community assembly. The unique multi‐habitat landscape of MF systems provides empirical evidence and new insights into ecosystem microbiome assembly.^[^ ^4^, ^12^ ^]^ At both local and global scales, we observed a positive relationship between habitat diversity and microbial diversity, a finding that echoes the “*Habitat heterogeneity hypothesis*” commonly applied to macroorganism biodiversity patterns.^[^ ^48^, ^52^ ^]^ Notably, research on macroorganisms typically assesses species richness within large areas and emphasizes the niche differentiation mechanisms. In contrast, our research relies on samples collected from localized sites and focuses on inter‐habitat connectivity. In other words, this study highlights that ecosystem‐level integration is crucial for deciphering microbial diversity distribution patterns and community assembly mechanisms. Our results also support the mass effect, wherein microbial populations in unfavorable habitats are maintained through continuous immigration from source communities, namely source‐sink dynamics.^[^ ^62^ ^]^ This process enables transient or less sustainable species to contribute to microbial richness and functionality.^[^ ^31^ ^]^ Microbial populations in one habitat can supply species pools for other habitats.^[^ ^63^ ^]^ As a result, greater habitat diversity increases the likelihood of diverse microbes dispersing into and enriching the local microbial community within each habitat. The classification of nonnative taxa can be confounded by temporal dynamics or detection thresholds. Variations in environmental conditions across habitats may also skew analyses and underestimate the contributions of putative nonnative species. For instance, soil's complex structure and micro‐habitat can promote species coexistence.^[^ ^64^ ^]^ Despite the inevitable presence of false positives and false negatives, our analyses consistently underscore the critical role of multi‐habitat landscapes in shaping ecosystem microbiomes. These findings align with the recently proposed “*Unified hypothesis*”, which posits dispersal‐based processes as critical biodiversity maintenance mechanisms.^[^ ^12^ ^]^ While our MAG‐level analysis offers valuable insights, it may be limited by the underestimation of low‐abundance microorganisms and strain‐level variations. To fully elucidate the ecological importance of putative nonnative taxa, further investigations should employ complementary approaches including complete genome references, ultradeep metagenomic sequencing, and synthetic microbial consortia.^[^ ^65^, ^66^ ^]^ Additionally, habitat diversity inferred from remote sensing could be confounded by resolution limitations, geographic variability, and climatic factors.^[^ ^67^ ^]^ Future research should examine the direct and indirect contributions of global habitat diversity to microbial diversity. Collectively, this study paves the way for ecosystem‐wide microbial assembly research, highlighting the need to integrate multi‐habitat perspectives into microbiome studies. The implications of our results extend to conservation policy, advocating for strategies that prioritize habitat diversity to safeguard microbiome health and ecological functionality worldwide. In agricultural contexts, habitat diversity can be achieved through various sustainable practices, such as crop rotation (temporal heterogeneity), intercropping, and the establishment of seminatural habitats,^[^ ^68^, ^69^ ^]^ which could enhance the sustainability and resilience of agricultural ecosystems, and ensure food production.

Inter‐habitat connectivity within ecosystems facilitates cross‐habitat taxa while driving evolutionary selection pressure that favors microbes adaptable to diverse environments. Notably, the ecological and functional convergences observed among cross‐habitat microbial taxa shed light on their adaptive strategies in diverse environments. These taxa exhibit pronounced microdiversity, characterized by genetically similar subtaxa, supporting their broader niches across habitats.^[^ ^36^ ^]^ Such microdiversity could enhance the resilience of microbial functionality under fluctuating environmental conditions,^[^ ^70^ ^]^ thereby stabilizing the ecosystem functions that they mediate. On the other hand, their widespread presence across habitats is underpinned by genetic adaptations that enable survival in varying environmental conditions. One notable trait is their higher number of 16S rRNA operon copies, which facilitates rapid growth and swift responses to resource availability—critical for organisms navigating multiple habitats with dynamic conditions. Genomic enrichment in functions related to genetic information processing, such as non‐homologous end‐joining (an error‐prone DNA repair process) and ribosome activity, aligns with the observed microdiversity and capability for rapid growth rates. Cross‐habitat taxa also exhibit enrichment in metabolism‐related pathways, including cofactor synthesis and amino acid synthesis. This allows them to sustain life in resource‐variable environments and exploit diverse ecological niches without relying on stable cross‐feeding relationships. Consistently, environmental variability could promote metabolic autonomy by reducing metabolic dependencies between microorganisms.^[^ ^71^, ^72^ ^]^ The trade‐off appears to be a reduction in habitat‐specific traits (e.g., biofilm formation and chemotaxis), which may be less critical for cross‐habitat taxa. Collectively, these patterns highlight the interplay between environmental variability and microbial adaptability,^[^ ^73^ ^]^ providing insights into the mechanisms that facilitate microbial success across diverse ecological contexts within multi‐habitat landscapes.^[^ ^74^ ^]^ Notably, these taxa span a wide range of taxonomic and phylogenetic lineages, suggesting a broader ecological strategy rather than a lineage‐specific phenomenon.

## 4 Conclusion

This study provides the first comprehensive analysis of microbial diversity within the MF agricultural heritage, revealing novel genetic resources. The insights gained from the MF system underscore the role of multi‐habitat landscapes in shaping microbiomes, as supported by global dataset analysis. Taxa found across multiple habitats exhibit convergence in microdiversity and adaptive genetic traits, revealing both ecological and functional mechanisms underlying their adaptability. These findings highlight the importance of ancestral ecological wisdom in developing sustainable agricultural strategies, enhancing microbial diversity, and reinforcing ecosystem resilience in the face of global change.

## 5 Experimental Section

### Sample Collection

The Huzhou MF systems, located in Taihu Lake Basin, have been designated as GIAHS.^[^ ^23^ ^]^ Samples were collected from 21 MF systems at the core conservation base region for the Huzhou MF Systems (30.763° N, 120.159° E; 30.700° N, 120.132° E), in Zhejiang Province, China during March, May, July, and December of 2021 and 2022. Six major habitats within the MF system, including water, sediment, soil, mulberry leaf, silkworm gut, and fish gut, were collected as previously described.^[^ ^60^ ^]^ Briefly, for water samples, a volume of 400 mL of the collected water was filtered through a 0.22 µm nylon filter (Millipore, USA). Sediment samples were collected in replicates within a radius of 1 m and pooled to yield a composite sample from each site.^[^ ^75^ ^]^ Soil samples were collected from bulk soil at least 50 cm away from plants, at a depth of 10 cm, and free of root or plant debris, as previously described.^[^ ^76^, ^77^ ^]^ Fresh mulberry leaves were collected in triplicate from individual plants. To minimize contamination from host sequences, epiphytes were collected using sonication, vortexing, and filtration through a 0.22 µm nylon filter, following established protocols.^[^ ^78^ ^]^ The silkworm hybrid strain “Mercury 1” provided by Huzhou Academy of Agricultural Sciences was reared using fresh mulberry leaves collected from MF. The gut contents of silkworm larvae on the second day of the fifth instar were collected. Fish gut samples were collected from eight major fish species within the MF systems, including *Ctenopharyngodon idella, Hypophthalmichthys nobilis*, *Hypophthalmichthys molitrix, Pseudorasbora parva, Carassius auratus, Hemiculter leucisculus, Mylopharyngodon piceus*, and *Pelteobagrus fulvidraco*. The fish specimens were anesthetized by freezing before being dissected to obtain gut contents. Four individuals from each species were collected. After DNA extraction, quality control, and sample pairing between bacterial and fungal amplicon data described below, the final numbers of biological samples used for each fish species were 1, 1, 3, 4, 4, 1, 1, and 3, respectively. All obtained samples were frozen using liquid nitrogen and subsequently stored at −80 °C. For comparative analysis, soil samples were additionally collected from normal mulberry fields across seven major sericulture production provinces in China: Zhejiang, Jiangsu, Guangdong, Guangxi, Chongqing, Sichuan, and Yunnan (*n* = 3, 5, 2, 3, 7, 3, and 5). These normal mulberry fields were only used for conventional mulberry planting without integration with other habitats in fishponds (e.g., pond water irrigation or sediment as fertilizers). All samples underwent identical processing protocols to ensure methodological consistency. Genomic DNA was extracted using the DNeasy PowerSoil Pro Kit (QIAGEN, Germany), following the manufacturer's instructions. Total RNA was extracted from water, soil, and sediment samples using the Soil RNA Extraction Kit (Majorbio, China) following the manufacturer's procedure. The integrity and quantity of the extracted nucleic acids were measured with a NanoDrop 2000 spectrophotometer (Thermo Scientific, USA) and an Agilent 5300 Bioanalyzer (Agilent Technologies, USA).

### Amplicon Sequencing and Processing

To identify the constituents of the communities, marker genes were amplified by PCR from community genomic DNA using universal primers including 515F_modF and 806R_modR, targeting the 16S rRNA gene V4 region of bacteria; ITS1F and ITS2R, targeting the internal transcribed spacer of fungi, as previously described.^[^ ^79^ ^]^ For V4 amplification, a mixture of peptide nucleic acid (PNA) blocker oligos (PNA Bio, USA) was added to inhibit the amplification of mitochondrial (mPNA) and chloroplast (pPNA) 16S rRNA genes without biasing results.^[^ ^80^ ^]^ The Illumina sequencing library was prepared and then sequenced on an Illumina NextSeq (PE250), following the manufacturer's instructions provided by Majorbio (Shanghai, China). Template‐free water blanks were processed with the same procedure as the negative controls to check for reagent and laboratory contamination.^[^ ^81^ ^]^ The paired‐end reads generated from Illumina sequencing were processed using the LotuS2 pipeline v2.25 with strict filtering criteria by performing quality filtering, ITSx (for fungi), binomial error model, dada2 denoising, chimera removing, and LULU reclustering with default parameters to generate curated and refined ASVs.^[^ ^82^ ^]^ Additionally, frequency‐based decontamination was performed to remove potential contamination (false discovery rate‐adjusted *P* < 0.05) using R package decontam v1.18.^[^ ^83^ ^]^ Taxonomic assignment of bacteria and fungi was performed based on SILVA v138.1 and UNITE v9.0 (including fungi and other eukaryotes) databases^[^ ^84^, ^85^ ^]^ using a naive Bayes consensus taxonomy classifier, with default parameters.^[^ ^86^ ^]^ To avoid influences by non‐target amplification and sequence artifacts, only ASVs identified as bacteria in 16S rRNA amplicon data and fungi in ITS amplicon data were kept for the following analysis. Samples lacking either bacterial 16S rRNA gene amplicon data or fungal ITS amplicon data were excluded from the analyses. Consequently, a total of 830 paired amplicon data (415 bacteria and 415 fungal amplicon data) were obtained from water, sediment, soil, mulberry leaf, silkworm gut, and fish gut samples: *n* = 99, 52, 157, 77, 12, and 18, respectively (Table S2, Supporting Information). To retain sufficient reads for characterizing microbial diversity in each habitat type, the number of sequences was rarefied to the minimum sequencing depth in each habitat:^[^ ^87^ ^]^ 34252, 42648, 49766, 2137, 18295, 38212 (bacterial amplicon), and 3842, 11363, 19472, 38650, 58402, 9077 (fungal amplicon) for water, sediment, soil, mulberry leaf, silkworm gut, and fish gut, respectively. The bacterial and fungal amplicon data of normal mulberry field soil were rarefied to 49766 and 38650 to align with the depth of MF soil amplicon to minimize the effect of sequencing depth on alpha diversity comparison. Phylotypes that are classified as bacteria/fungi using taxonomic databases but do not match data at the phylum level are expected to be potential new phyla.^[^ ^24^ ^]^ To compare the abundance of phylum‐level unclassified phylotypes between MF soil, normal mulberry field soil, and global soil from Delgado‐Baquerizo's work (Illumina MiSeq platform, 10 000 rarefaction depth, and OTU‐resolved bioinformatics pipelines),^[^ ^24^ ^]^ data were rarefied to 10 000 and all phylotypes were re‐annotated based on Greengenes v13.8 and UNITE v8.0 for bacteria and fungi, respectively, using the naive Bayes consensus taxonomy classifier as previously described.^[^ ^24^, ^85^, ^86^, ^88^ ^]^ Since the ratio of unknown phylotypes might be biased by data processing (such as generation of OTU and ASV), the relative abundance was used to compare the unknown phylotypes between samples and datasets.^[^ ^24^ ^]^ To visualize the broad taxonomic distribution of cross‐habitat taxa, the representative ASVs that were present in all six habitats were highlighted on phylogenetic trees constructed using the FastTree plugin in QIIME2 v2022.10 with default parameters.^[^ ^86^ ^]^ The 16S rRNA operon counts of ASVs were estimated using rrnDB v5.8 based on their closest relatives with known rRNA operon copy numbers.^[^ ^89^ ^]^

### Metagenomic and Metatranscriptomic Sequencing and Processing

Shotgun metagenomic sequencing was performed on water, sediment, soil, mulberry leaf, and fish gut samples (*n* = 28, 27, 28, 16, and 9, respectively; Table S2, Supporting Information). The silkworm gut was excluded due to technical difficulties in obtaining microbial reads caused by nucleic acid contamination from the plant leaf diet. After sequencing and quality control, the fish gut samples from *Ctenopharyngodon idella, Hypophthalmichthys nobilis, Hypophthalmichthys molitrix, Carassius auratus, and Hemiculter leucisculus* were used for metagenomic analyses, *n* = 1, 1, 4, 2, and 1, respectively. Metatranscriptomic sequencing was performed for three environmental habitats, including water, sediment, and soil (*n* = 10, 8, and 6, respectively; Table S2, Supporting Information). The RNA was subjected to standard Illumina library preparation with an Illumina Stranded mRNA Prep, Ligation (Illumina, USA), and rRNA was depleted using a RiboCop rRNA Depletion Kit (Lexogen, USA). Sequencing was both performed on the Illumina Novaseq platform (PE150). Quality control was performed using fastp v0.23.2, and potential host‐derived reads were removed using Bowtie2 v2.5.4.^[^ ^79^ ^]^ Non‐coding RNA sequences were removed using SortMeRNA v4.3.6 with default parameters.^[^ ^90^ ^]^ Consequently, a total of 1620.3 Gbp clean metagenomic reads and 110.4 Gbp clean metatranscriptomic reads were obtained.

Additionally, 1182 nonredundant MAGs (dereplicated at a 95% similarity cutoff, approximately at the species level) were obtained from the gene and genome catalog of the MF system (MF2G) (https://doi.org/10.5281/zenodo.13147328), which was assembled and binned from the 108 metagenomic data. Briefly, MAGs were constructed by combining binning methods, including MetaBAT2 v2.12.1, metadecoder v1.0.16, binny v3.0.9, and SemiBin2 v2.0.2,^[^ ^91^, ^92^, ^93^, ^94^ ^]^ as previously described.^[^ ^95^ ^]^ The resulting bins were then aggregated, refined, and dereprelicated at the 0.95 similarity cutoff.^[^ ^95^ ^]^ The quality of the resulting MAGs was evaluated using CheckM v1.2.2 to retain medium‐quality MAGs (>50% completeness and <10% contamination).^[^ ^96^ ^]^ These MAGs have an average completeness of 76.93% (±0.79%) and a contamination rate of 3.11% (±0.13%). The maximal average nucleotide identity values of genomes from the global soil genomic catalog (*n* = 40039)^[^ ^25^ ^]^ and MF soil (*n* = 218) were calculated by comparison against GTDB r207 reference genomes, as previously described.^[^ ^97^ ^]^ The relative abundance or expression of each MAG in the metagenome/metatranscriptome was calculated using CoverM v0.6.1 as previously described.^[^ ^61^ ^]^ Genomic annotation was performed using the eggNOG database v5.0 and eggnog‐mapper v2.1.12 with default parameters.^[^ ^98^ ^]^ Minimal doubling times were predicted from the codon usage statistics of MAG using gRodon v2.3.0.^[^ ^42^ ^]^

### Analysis of Putative Nonnative Taxa

The niche breadth was estimated using Levins' niche breadth index,^[^ ^99^ ^]^ described by the formula:

(1) $$B_{j}=1/\sum_{i=1}^{N}P_{ij}^{2}$$

where *B_j_* indicates niche breadth, and *P_ij_* is the proportion of individuals belonging to taxa *j* present in a given community *i*. Taxa that are present and more evenly distributed along a wider range of habitats have a higher *B*‐value and can be considered habitat generalists.^[^ ^100^, ^101^ ^]^ Taxa with a lower *B*‐value can be regarded as habitat specialists. To compare the niche breadth of taxa across habitats, niche breadth was normalized by dividing the maximal niche breadth index within each habitat. By integrating all six major habitats within the MF system, potential nonnative taxa were predicted based on their occurrence, including relative abundance and niche breadth index. Specifically, for each pair of habitats (*H*) and species, species were categorized as putative nonnative only when species exhibited: lower relative abundance than in other habitats (other than *H*); lower niche breadth index than indices in other habitats (other than *H*); lower niche breadth index than indices calculated from other species in the habitat *H*. At the community level, the taxonomic and functional diversity of observed and simulated communities was evaluated by including or excluding their presence. The functional richness was assessed based on the KEGG and COG annotation of MAGs. The functional redundancy index was calculated based on the phylogenetic distribution of community members, namely, MAGs, harboring specific functions.^[^ ^102^ ^]^ In the cases, carbon, nitrogen, phosphorus, and sulfur cycling‐related biogeochemical functions were characterized based on KEGG annotation. The source tracking analysis was performed using SNV‐FEAST,^[^ ^33^ ^]^ which relies on the identification of single nucleotide variants within metagenomic datasets. BWA‐MEM v0.7.17 was used to map high‐quality sequencing reads against genome sequences. Variants were identified using Metapop v2022^[^ ^103^ ^]^ with parameters: –minimum_bases_for_detection 3000 –min_obs 1 –min_cov 10 –min_pct 0 –no_viz. To ensure sufficient variant detection, a strategy of combining metagenomes from each habitat was employed using SAMtools v1.19.^[^ ^104^ ^]^

### Global Habitat Heterogeneity Analysis

The spatial heterogeneity of the global habitat was evaluated using Global Habitat Heterogeneity metrics based on the textural features of EVI imagery (250 m spatial resolution) acquired by the Moderate Resolution Imaging Spectroradiometer (MODIS), as previously described.^[^ ^49^ ^]^ The Shannon metric, which measures first‐order texture (compositional variability at a 1 km spatial resolution) in EVI, was used to estimate habitat heterogeneity (https://www.earthenv.org/texture).^[^ ^49^ ^]^ Higher values indicate higher habitat heterogeneity within the region. The habitat heterogeneity index was retrieved using raster v3.6‐23 based on the recorded latitude and longitude from online datasets and was normalized using standard scaling. Global soil bacterial (Earth Microbiome Project) and fungal (Global Soil Mycobiome consortium) amplicon data were obtained from the publicly available amplicon sequencing resources (Table S3, Supporting Information) since they cover a wide range of sampling sites at a global scale.^[^ ^54^, ^55^ ^]^ The abundance tables were rarefied to 2000 to minimize the impact of sequencing depth. The taxonomic diversity of bacterial and fungal communities was calculated using the Shannon index. Global soil metagenomic data were downloaded from the Sequence Read Archive based on previously collected accession numbers.^[^ ^25^ ^]^ Soil metagenome samples from deep soil (> 20 cm) were excluded from the analysis. Accession numbers of public soil metagenomes used in this study are provided in Table S3 (Supporting Information). Functional diversity was calculated at a depth of 1 000 000 reads based on the metagenomic diversity index^[^ ^105^ ^]^ using a Python script modified from https://github.com/DamienFinn/MD/blob/main/MD.py. The key climatic factors, including mean annual temperature and mean annual precipitation,^[^ ^106^, ^107^ ^]^ were obtained from the WorldClim v2.1 database. The correlations between habitat heterogeneity and microbial diversity were calculated based on linear mixed models controlling study identity, land type, and climatic factors using lme4 v1.1‐35.2.

### Microdiversity and Genetic Enrichment Analysis

The microdiversity of cross‐habitat taxon was assessed at the OTU and ASV level using two methods: 1) the number of ASVs within belonging OTUs (intra‐OTU ASV number), and 2) the nearest taxon distance, which measures the phylogenetic distance between a given taxon and its closest relative within the dataset (minus log‐transformed to represent inter‐ASV closeness), as previously described.^[^ ^36^ ^]^ OTUs were clustered from ASVs at a 0.97 similarity cutoff using vsearch v2.22.1 with the parameters –cluster_size –id 0.97.^[^ ^108^ ^]^ Higher intra‐OTU ASV number and inter‐ASV closeness suggest greater microdiversity. The microdiversity of taxa that occur in different numbers of habitat types was calculated and compared. The microdiversity of taxa at a global scale was also analyzed using amplicon data from the Earth Microbiome Project^[^ ^54^ ^]^ and samples were categorized into six habitat types, including animal, plant, soil, water, sediment, and dust, according to the recorded metadata. To analyze the genetic mechanisms underlying the cross‐habitat taxa, namely occurring in multiple habitats, microbial functional enrichment analysis was performed based on the gene composition of MAGs, using ReporterScore v0.1.4.^[^ ^109^ ^]^ Specifically, KEGG gene annotations were used to reveal the functional genes that are carried by MAGs that were present in multiple habitats. The c‐means clustering was conducted to identify clusters of encoded genes by MAGs that occur in different numbers of habitat types, using “*RSA_by_cm*” function, and plotted using “*plot_c_means*” function. Then, these clusters were used for Generalized Reporter Score‐based Analysis (GRSA) to obtain and visualize enriched pathways, using “*plot_report_bar*” function.

### Statistical Analysis

All statistical analyses were conducted using R v4.2.2.^[^ ^110^ ^]^ Normal distribution and homoscedasticity were assessed by the Shapiro–Wilk and Levene's test, respectively. Data were presented as mean ± standard error of the mean. Comparisons of relative abundance and diversity between groups were performed using the t‐test or nonparametric rank‐sum test. A two‐sided *P* value of <0.05 was considered statistically significant. NMDS was performed using Bray‐Curtis dissimilarity. PERMANOVA was performed to disentangle variation across habitats, MF identities, conservation base regions, and seasons, using Bray‐Curtis dissimilarity with 9999 permutations. Null model‐based analysis of assembly processes was performed across habitats using iCAMP v1.6.1.^[^ ^111^ ^]^ The number of shared and unique ASVs between habitats was visualized using the Venn plot and the UpSet plot.

### Ethics Approval and Consent to Participate

All procedures involving fish experiments were approved by the Ethical Committee of Zhejiang University (Approval number ZJU20250588).

## Conflict of Interest

The authors declare no conflict of interest.

## Author Contributions

J.H. and Y.S. designed the overall study. J.H., J.X., X.S., and Y.S. collected the samples. J.H. performed the bioinformatic analyses. J.H. wrote the first draft manuscript. All authors aided in editing the manuscript and approved the final manuscript.

## Supporting information



Supporting Information

## Data Availability

The data that support the findings of this study are openly available in NCBI at https://www.ncbi.nlm.nih.gov/bioproject/?term=PRJNA881590, reference number 881590.
