# Supplementary material for: Multi‐Habitat Landscape Promotes Microbial Diversity: Insights from the Traditional Agricultural Heritage and the Global Trend
Source: Adv Sci (Weinh). 2025 Sep 23;12(46):e06402. doi: 10.1002/advs.202506402 (PMC12697846; doi:10.1002/advs.202506402)
Supplement: Supplementary file 1 — Supporting Information [file ADVS-12-e06402-s001.docx]

***Supporting Information for***

**Multi-habitat Landscape Promotes Microbial Diversity: Insights from the Traditional Agricultural Heritage and the Global Trend**

*Jintao He, Jian Xiao, Xiaoqiang Shen, Kankan Zhao, Xiaoyu Lei, Huarui Zhang, Chao Sun, Huijie Lu, Yongqi Shao**

Y. Shao

Zhejiang University, Hangzhou 310000, China; Key Laboratory of Silkworm and Bee Resource Utilization and Innovation of Zhejiang Province, Hangzhou, China; Key Laboratory for Molecular Animal Nutrition, Ministry of Education, Hangzhou, China

E-mail: [yshao@zju.edu.cn](mailto:yshao@zju.edu.cn)

J. He, J. Xiao, X. Shen, X. Lei, H. Zhang, Y. Shao

Max Planck Partner Group, Institute of Sericulture and Apiculture, Faculty of Agriculture, Life and Environmental Sciences, Zhejiang University, Hangzhou, China;

K. Zhao

Institute of Soil and Water Resources and Environmental Science, College of Environmental and Resource Sciences, Zhejiang University, Hangzhou, China

C. Sun

Analysis Center of Agrobiology and Environmental Sciences, Zhejiang University, Hangzhou, China

H. Lu

Key Laboratory of Environmental Remediation and Ecological Health, Ministry of Education, College of Environmental and Resource Sciences, Zhejiang University, Hangzhou, China; Key Laboratory of Water Pollution Control and Environmental Safety, Zhejiang, China

# Supplementary Figures


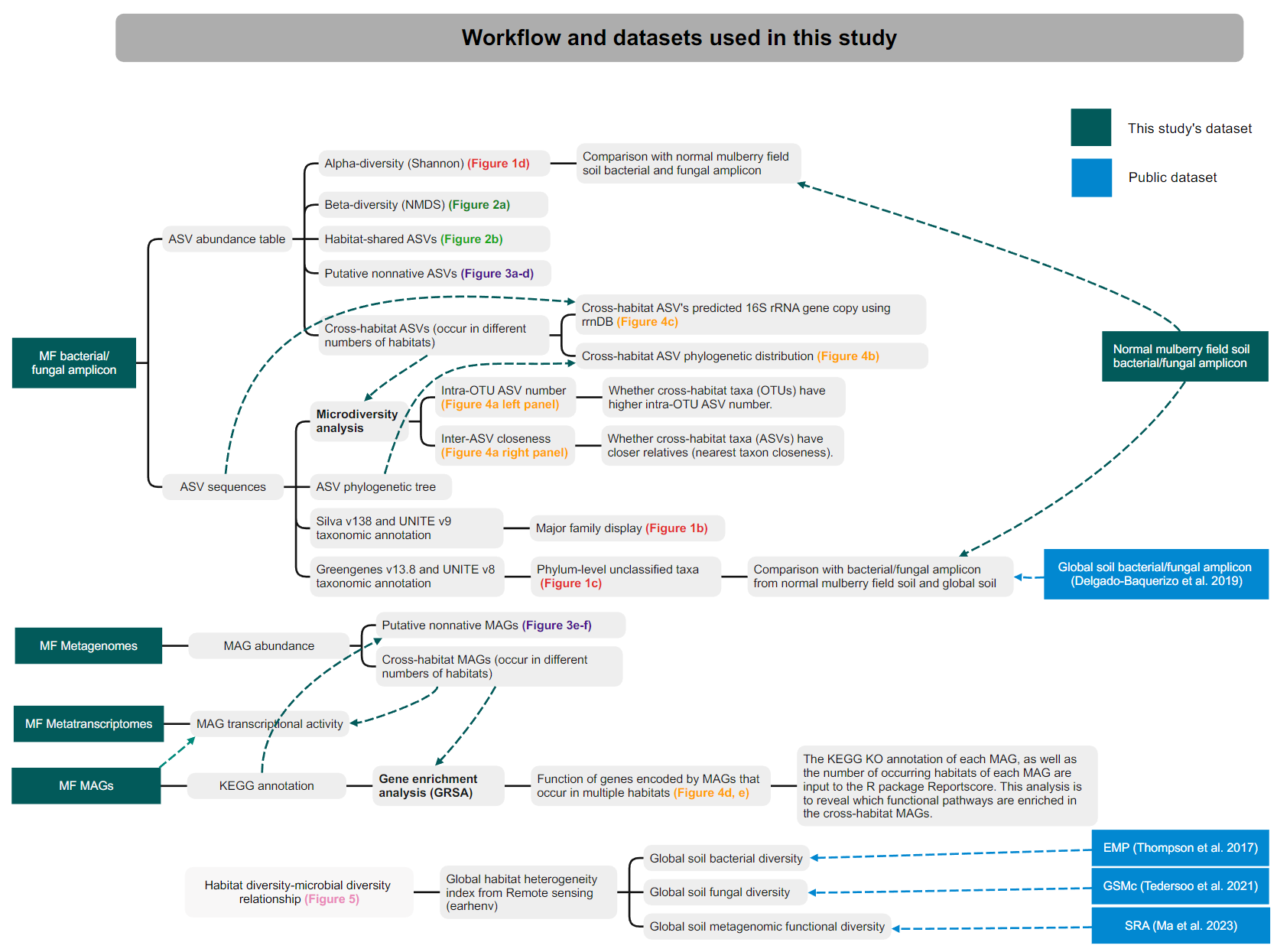


1. The workflow and datasets used in this study. The colored boxes discriminate the datasets from our study (dark green) and the public databases (blue). White boxes indicate the analyses performed to generate the Main Figures. Dashed arrow lines indicate that the datasets or results were used as input to perform pointed analysis.

*
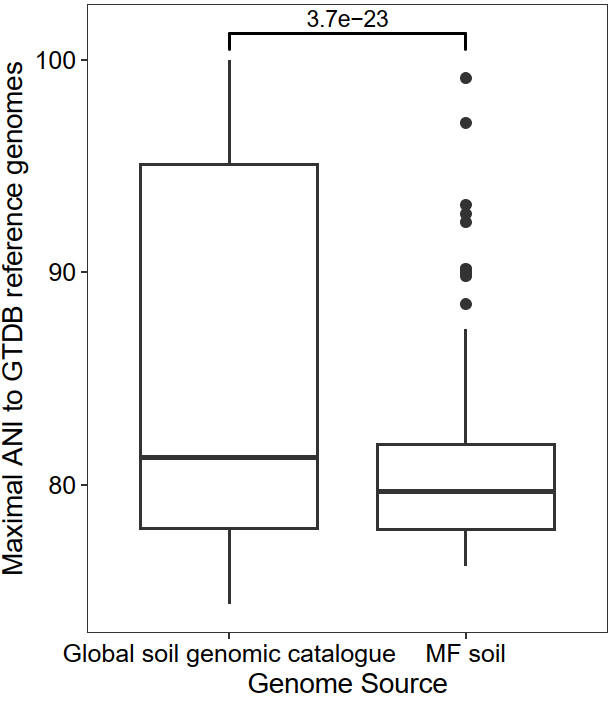
*

1. The maximal Average Nucleotide Identity (ANI) of genomes from the global soil genomic catalog (n = 40,039) and MF soil (n = 218) to GTDB r207 reference genomes. Lower maximal ANI value indicates lower similarity to reference genomes, thereby indicating novelty, as previously described (Levin D et al. 2021). The global soil genomes were retrieved from the work of Ma B et al. in 2023. *P* value calculated by Welch’s t-test was shown. Boxplots show the median (line), 25th and 75th percentiles (box), and 1.5 × the interquartile range (whiskers). Outliers are represented by dots.

*References*

*Ma B, Lu C, Wang Y, Yu J, Zhao K, Xue R, et al. A genomic catalogue of soil microbiomes boosts mining of biodiversity and genetic resources. Nat. Commun. 14 (2023) 7318.*

*Levin D, Raab N, Pinto Y, Rothschild D, Zanir G, Godneva A, et al. Diversity and functional landscapes in the microbiota of animals in the wild. Science 372 (2021) eabb5352.*


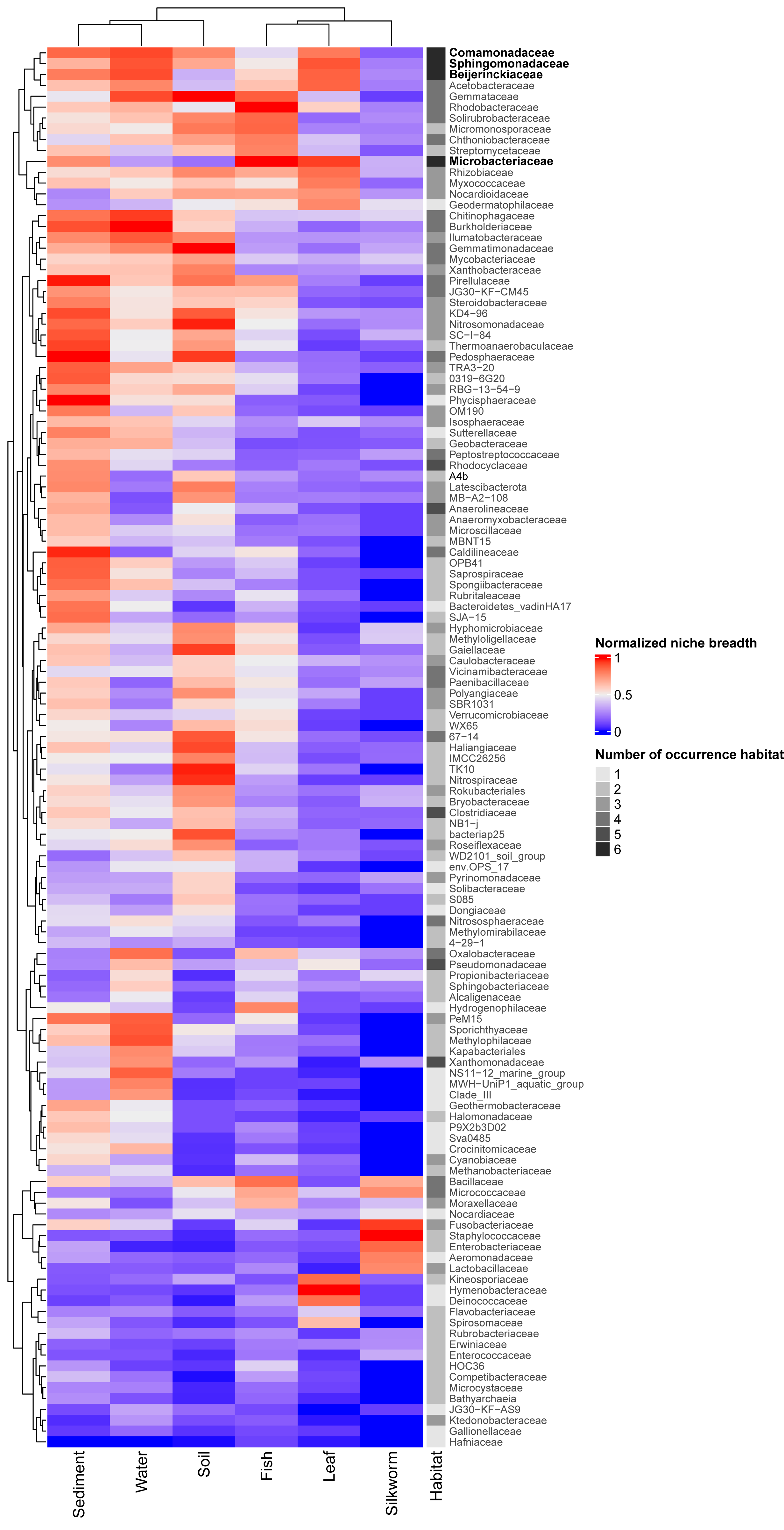


1. Heatmap depicting normalized niche breadth values of major bacterial families across six habitats. Representative families exhibiting broad habitat distribution (present in all six habitats), are highlighted in bold.


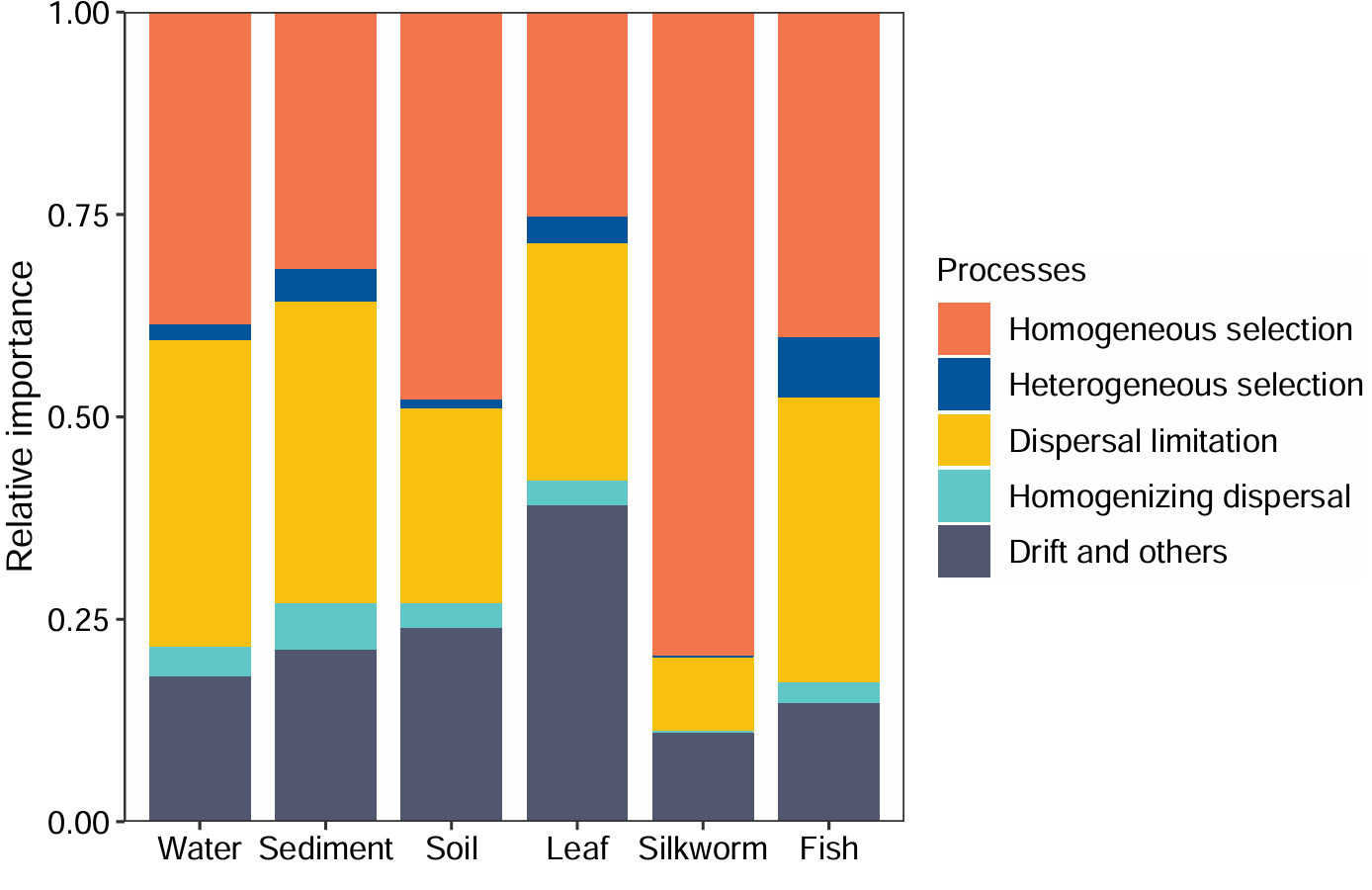


1. Assembly mechanisms of microbial communities across habitats.


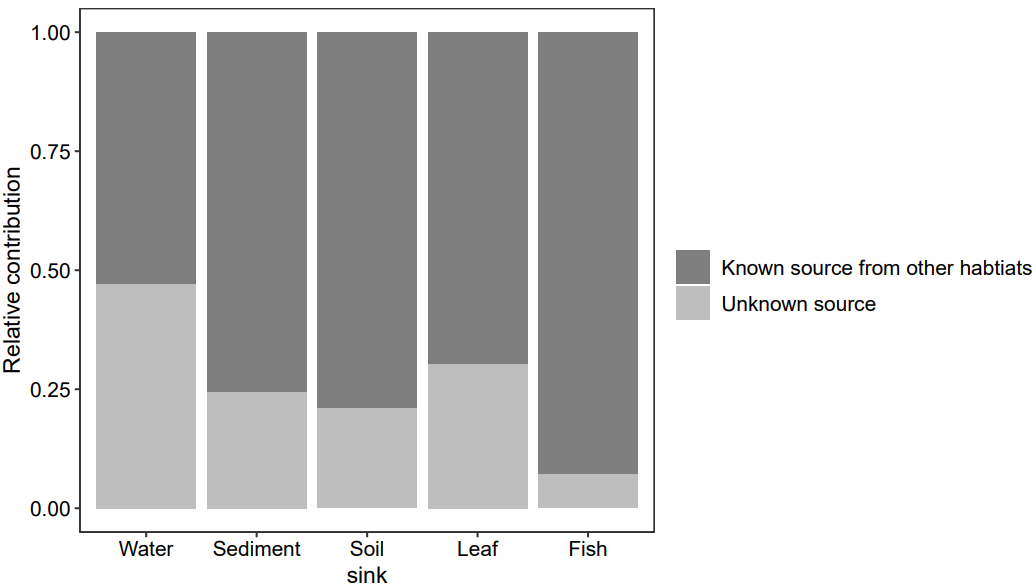


1. Explained variation of SNVs by putative nonnative taxa within available metagenomes from each habitat. The silkworm gut habitat was excluded from the analysis because technical issues in obtaining microbial reads resulted from significant host contamination.


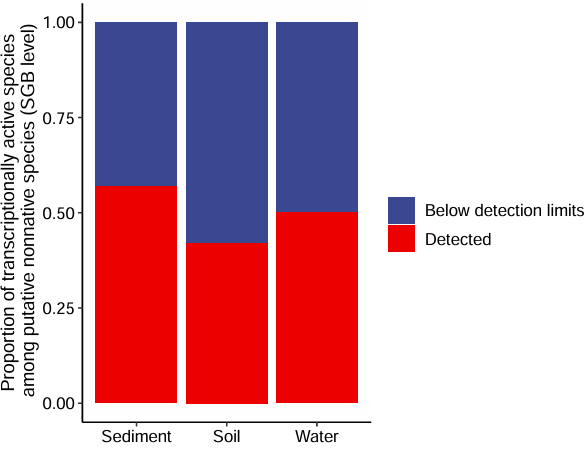


1. Proportion of transcriptionally active species among putative nonnative species in meta-transcriptomes. Sediment, soil, and water were subjected to metatranscriptomics (n = 8, 6, and 10, respectively) while host-associated habitats were excluded from the analysis due to host contamination. The analysis of transcriptional activity was conducted using bwa-mem mapping high-quality meta-transcriptomic reads against genomes.


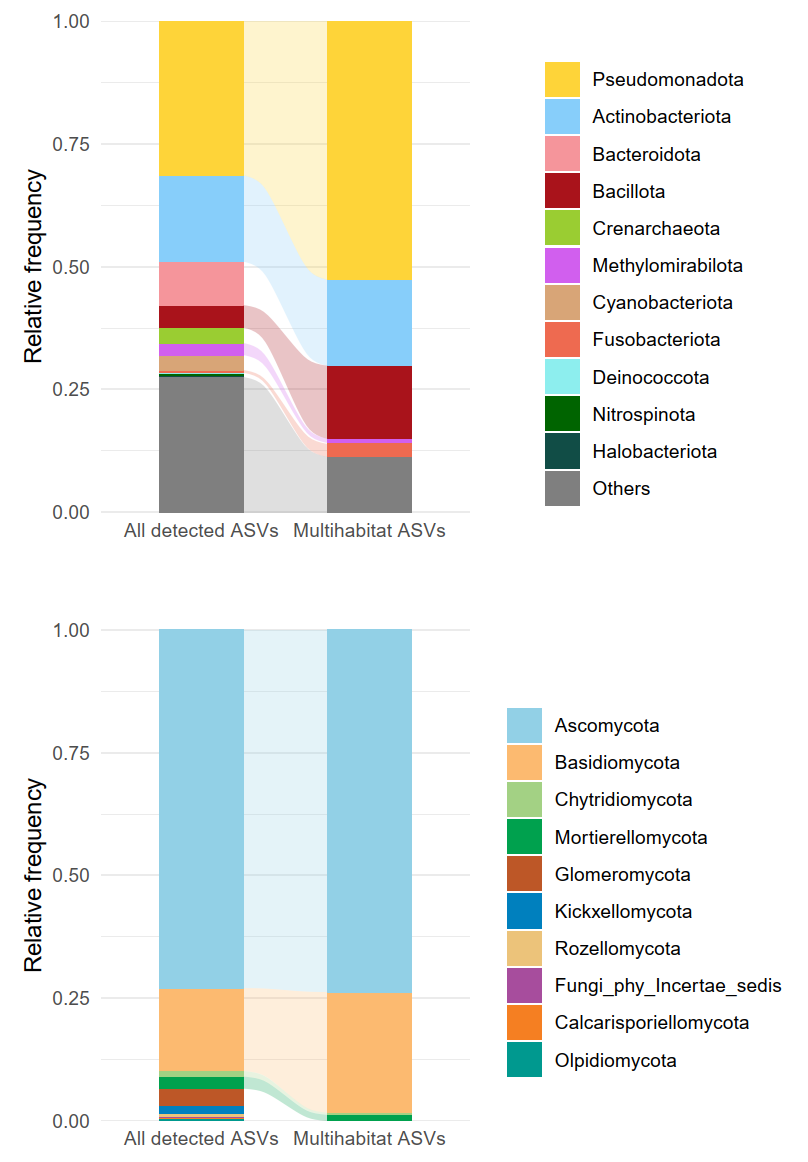


1. Phylum-level annotation of taxa occurring in all habitats. All detected ASVs represent all ASVs obtained from amplicon sequencing (n = 46,811 and 9,445 for bacteria and fungi, respectively). Multihabitat ASVs represent the ASVs (n =257 and 384 for bacteria and fungi, respectively) occurring in all habitats.


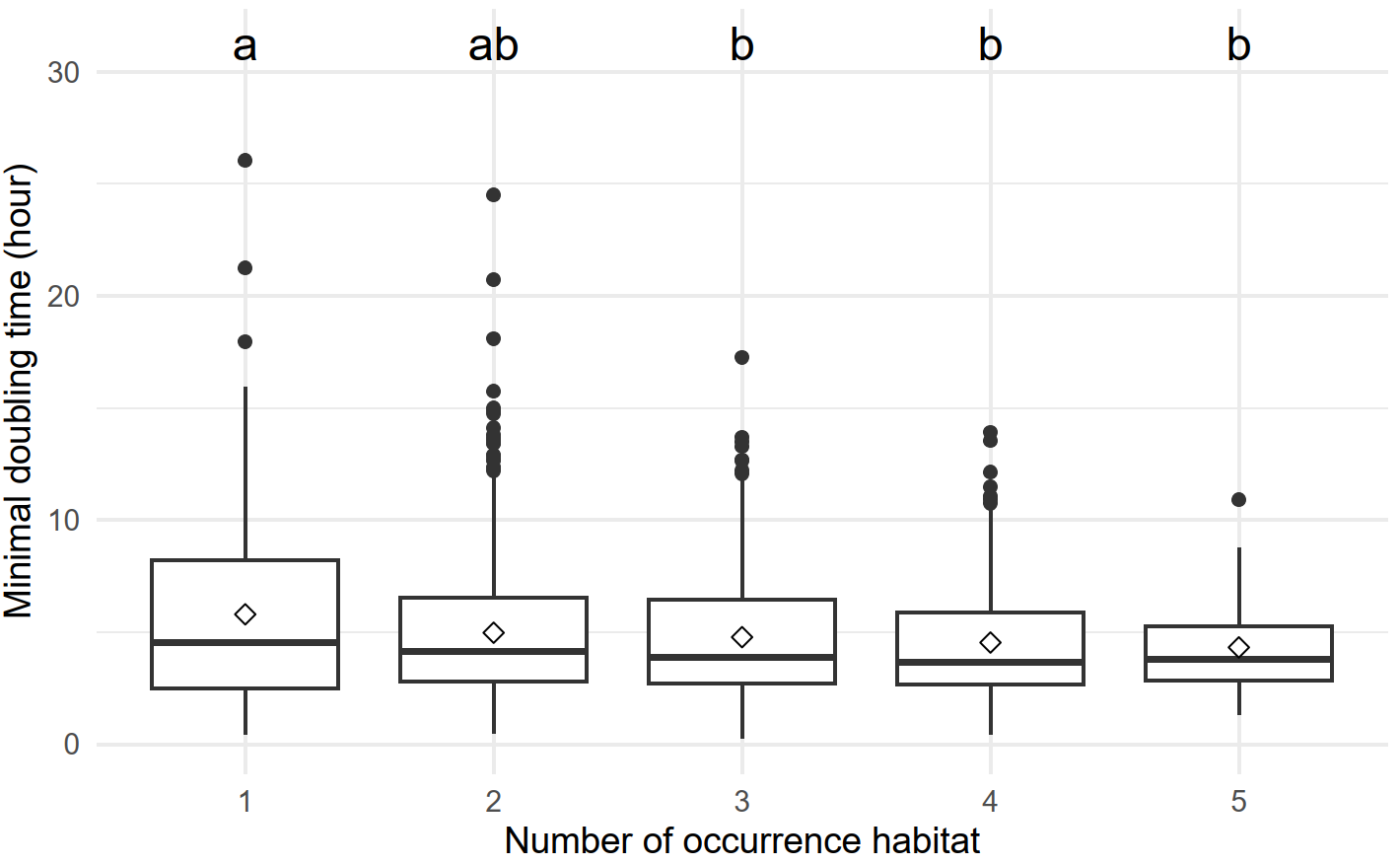


1. Minimal doubling times of MAGs with different numbers of occurrence habitats. Rhombus represents the mean value in each group. Higher values of minimal doubling times represent low maximal growth rates. n = 174, 556, 277, 142, and 33 (number of occurrence habitat from 1 to 5). Boxplots show the median (line), 25th and 75th percentiles (box), and 1.5 × the interquartile range (whiskers). Outliers are represented by dots. Groups that do not share a letter are significantly different (false discovery rate-adjusted *P* < 0.05, pair-wise t-test).


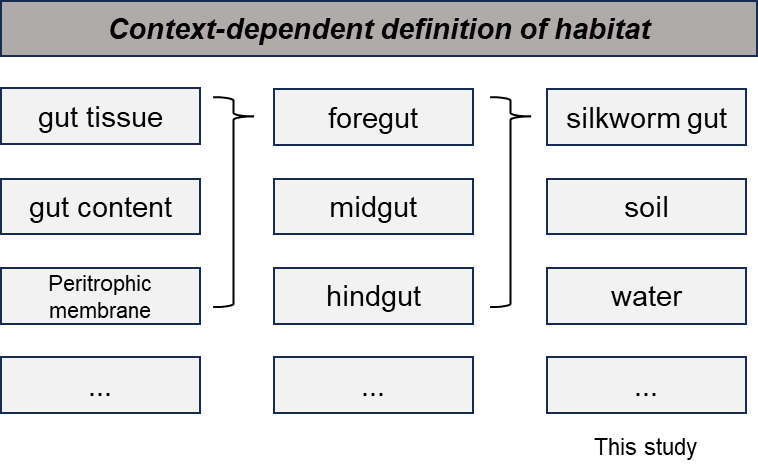


1. Schematic diagram showing a case of context-dependent definition of habitat.


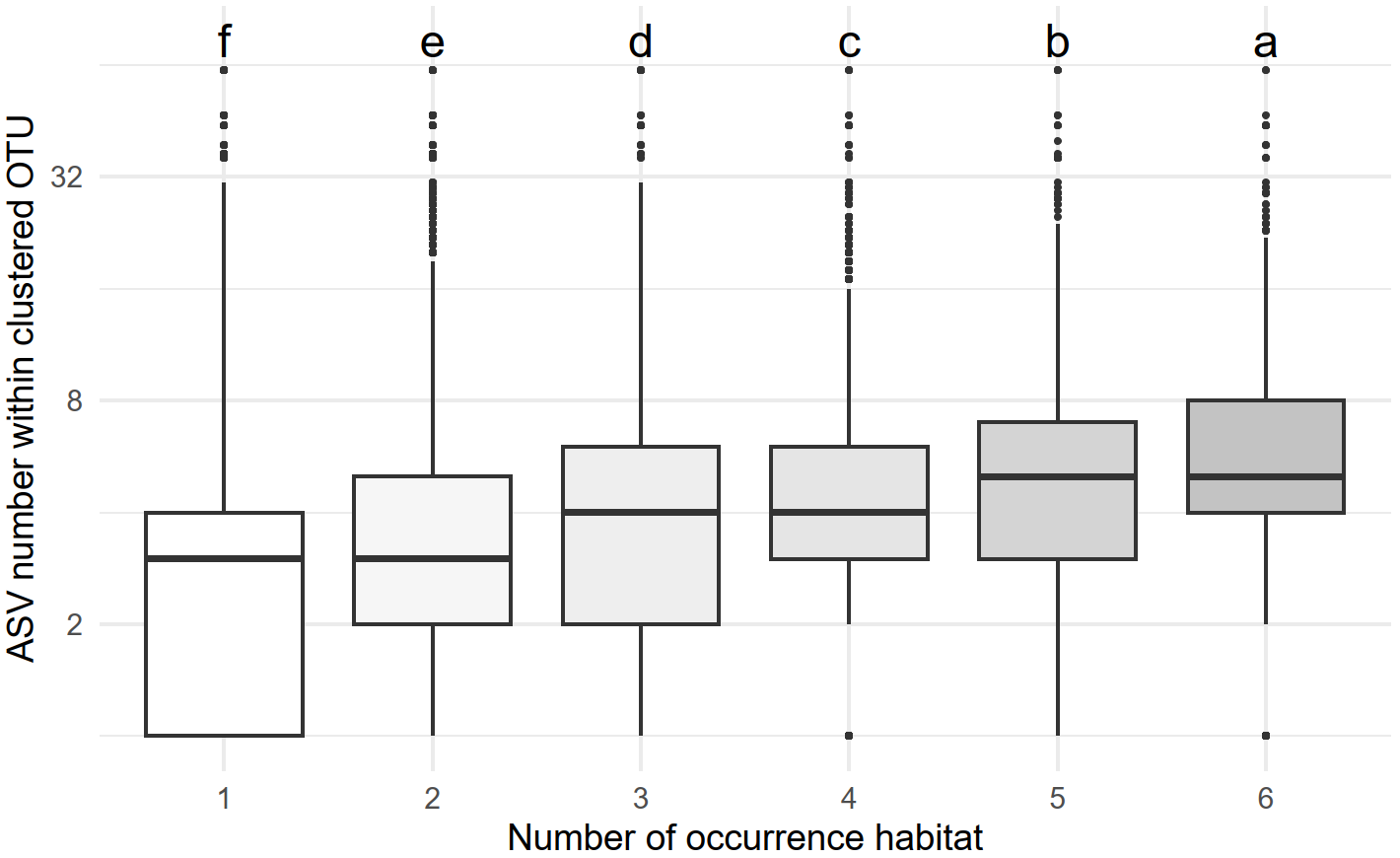


1. Higher microdiversity of taxa associated with multiple habitats at the global scale. Boxplot shows the ASV number within clustered OTU with different numbers of occurrence habitats. Higher values indicate higher microdiversity. n = 120,002, 80,100, 43,908, 23,412, 11,794 and 7,662 (number of occurrence habitat from 1 to 6). Boxplots show the median (line), 25th and 75th percentiles (box), and 1.5 × the interquartile range (whiskers). Outliers are represented by dots. Groups that do not share a letter are significantly different (false discovery rate-adjusted *P* < 0.05, pair-wise t-test).

# Supplementary Tables

1. Statistical results of linear mixed models.

|  | **Bacterial amplicon** | | | **Fungal amplicon** | | | **Metagenome** | | |
| --- | --- | --- | --- | --- | --- | --- | --- | --- | --- |
| *Predictors* | *Estimates* | *CI* | *p* | *Estimates* | *CI* | *p* | *Estimates* | *CI* | *p* |
| Habitat heterogeneity | 0.076208 | 0.018757 –  0.133659 | **0.009** | 0.063962 | 0.036160 –  0.091764 | **6.43e-06** | 0.009691 | 0.002429 –  0.016953 | **0.009** |
| MAT | -0.004771 | -0.026469 – | 0.666 | -0.001672 | -0.005955 – | 0.444 | 0.000086 | -0.001452 – | 0.913 |
|  |  | 0.016927 |  |  | 0.002611 |  |  | 0.001623 |  |
| MAP | -0.000288 | -0.000573 – | **0.048** | 0.000019 | -0.000063 – | 0.373 | 0.000006 | -0.000011 | 0.495 |
|  |  | -0.000003 |  |  | 0.000024 |  |  | – 0.000023 |  |

1. Overview of datasets used in this study.

| Datasets | | This study | | | | | | | Delgado-Baquerizo et al. | Thompson et al.  (EMP) | Tedersoo et al. (GSMc) | Ma et al. (SRA) |
| --- | --- | --- | --- | --- | --- | --- | --- | --- | --- | --- | --- | --- |
| Sample source | | MF system | | | | | | Normal mulberry | Global | Global | Global | Global |
|  |  |  |  |  |  |  |  | field |  |  |  |  |
| Investigated habitat | | Soil | Water | Sediment | Fish gut | Leaf | Silkworm gut | Soil | Soil | Soil | Soil | Soil |
| Bacteria | number | 157 | 99 | 52 | 18 | 77 | 12 | 28 | 235 | 1,989 | N/A | N/A |
| amplicon | major Figures | Figure 1, Figure 2, Figure 3a-d, Figure 4a-c | | | | | | Figure 1c-d | Figure 1c | Figure 5 | N/A | N/A |
| Fungi | number | 157 | 99 | 52 | 18 | 77 | 12 | 28 | 235 | N/A | 2,132 | N/A |
| amplicon | major Figures | Figure 1, Figure 2, Figure 3a-d, Figure 4a-c | | | | | | Figure 1c-d | Figure 1c | N/A | Figure 5 | N/A |
| Metagenome | number | 28 | 28 | 27 | 9 | 16 | N/A | N/A | N/A | N/A | N/A | 340 |
|  | major Figures | Figure3e-f | | | | | | N/A | N/A | N/A | N/A | Figure 5 |
| Metatranscriptome | number | 6 | 10 | 8 | N/A | N/A | N/A | N/A | N/A | N/A | N/A | N/A |
|  | major Figures | Figure S6 | | | | | | N/A | N/A | N/A | N/A | N/A |
| MAG | number | 218 | 539 | 339 | 52 | 34 | N/A | N/A | N/A | N/A | N/A | 40,039 |
|  | major Figures | Figure3e-f, Figure 4d-e | | | | | | N/A | N/A | N/A | N/A | Figure S2 |

Note: The Table shows the overview of datasets’ source (from this study or public database), omic type (bacterial amplicon, fungal amplicon, metagenome, metatranscriptome, MAG), numbers, and used major figures. “N/A” indicates not available. Amplicon data from Thompson et al. were not used in the comparison of microbial novelty since the sequences were trimmed to 90 bps, which could affect taxonomic annotation.

*References*

*M. Delgado-Baquerizo, Obscure soil microbes and where to find them, ISME J. 2019, 13, 2120.*

*L. R. Thompson, J. G. Sanders, D. McDonald, et al., A communal catalogue reveals Earth's multiscale microbial diversity, Nature 2017, 551, 457.*

*L. Tedersoo, V. Mikryukov, S. Anslan, et al., The Global Soil Mycobiome consortium dataset for boosting fungal diversity research, Fungal Divers. 2021, 111, 573.*

*B. Ma, C. Lu, Y. Wang, et al., A genomic catalogue of soil microbiomes boosts mining of biodiversity and genetic resources, Nat. Commun. 2023, 14, 7318.*

1. Accession number of public soil metagenomic samples used in this study.

| SRA Accession number | Longitude | Latitude |
| --- | --- | --- |
| DRR101455 | 119.1364 | 32.47979 |
| DRR160703 | 130.766 | 32.889 |
| DRR160715 | 140.244 | 38.249 |
| ERR1051325 | -97.5206 | 34.98167 |
| SRR10011679 | -147.84 | 64.85 |
| SRR10445787 | 104.16 | 25.263 |
| SRR10466945 | -123.631 | 39.7392 |
| SRR10857620 | 16.08 | -23.14 |
| SRR10857621 | 15.55 | -23.2 |
| SRR10968225 | 14.70483 | 48.66696 |
| SRR10968226 | 14.7088 | 48.66532 |
| SRR10968235 | 14.70579 | 48.66419 |
| SRR10968246 | 14.70931 | 48.66447 |
| SRR10968264 | 14.70447 | 48.66431 |
| SRR1153387 | -88.242 | 40.075 |
| SRR1157616 | -93.489 | 47.50505 |
| SRR1164896 | 117.5 | 38.7 |
| SRR1168459 | 117.5 | 38.7 |
| SRR1185960 | 111.4635 | 27.62182 |
| SRR12102596 | 34.97481 | 29.94152 |
| SRR12102597 | 34.76735 | 30.78534 |
| SRR12102598 | 34.74583 | 30.60917 |
| SRR12102600 | 34.91306 | 31.34917 |
| SRR12102602 | 34.75278 | 31.25333 |
| SRR12102603 | 34.91472 | 31.63028 |
| SRR12102604 | 34.94917 | 30.405 |
| SRR12102606 | 34.93304 | 31.64222 |
| SRR12102607 | 34.93501 | 31.64237 |
| SRR12284399 | 137.1363 | -30.4704 |
| SRR12284405 | -114.916 | 35.8171 |
| SRR12284406 | 96.2267 | 39.9022 |
| SRR12284407 | 15.5346 | -23.3107 |
| SRR12284408 | 137.1363 | -30.4704 |
| SRR12418375 | 144.4701 | -37.4464 |
| SRR12823103 | -119.737 | 46.2528 |
| SRR5915956 | 147.4597 | -42.2423 |
| SRR5915997 | 147.4226 | -42.6174 |
| SRR5916035 | 141.6164 | -13.8439 |
| SRR5916047 | 147.4561 | -42.5452 |
| SRR5916093 | 146.8927 | -42.2715 |
| SRR5916120 | 141.5062 | -13.7477 |
| SRR5916140 | 147.4223 | -42.6178 |
| SRR5916175 | 147.0474 | -42.3878 |
| SRR5916217 | 147.6145 | -42.279 |
| SRR5916275 | 147.3189 | -41.7775 |
| SRR5916285 | 145.0931 | -41.5837 |
| SRR5916294 | 147.3109 | -42.3843 |
| SRR5916354 | 147.4398 | -42.8072 |
| SRR5916420 | 147.4077 | -42.4042 |
| SRR5918911 | 131.0423 | -25.351 |
| SRR5918912 | 131.0423 | -25.351 |
| SRR5918916 | 145.4479 | -16.1034 |
| SRR5918921 | 145.6309 | -17.1211 |
| SRR5918944 | 140.7064 | -34.0372 |
| SRR5918954 | 146.6543 | -43.0951 |
| SRR5918955 | 135.7763 | -13.4082 |
| SRR5918958 | 144.0539 | -37.2521 |
| SRR5918960 | 140.3515 | -34.098 |
| SRR5918970 | 130.9286 | -25.2875 |
| SRR5918978 | 126.9831 | -31.8633 |
| SRR5918986 | 150.7353 | -35.153 |
| SRR5918988 | 131.085 | -25.3119 |
| SRR5918989 | 131.084 | -25.3118 |
| SRR5918992 | 141.9968 | -35.1228 |
| SRR5918993 | 142.2893 | -36.6715 |
| SRR5918994 | 142.2893 | -36.6715 |
| SRR5918997 | 141.9968 | -35.1228 |
| SRR5919098 | 133.2493 | -22.2828 |
| SRR5919101 | 134.5062 | -13.5212 |
| SRR5919102 | 135.8103 | -12.5092 |
| SRR5919105 | 145.0292 | -36.6732 |
| SRR5919106 | 141.9763 | -35.1164 |
| SRR5919168 | 150.6863 | -35.1497 |
| SRR5919197 | 131.0516 | -25.3504 |
| SRR5919198 | 131.0516 | -25.3504 |
| SRR5919221 | 134.6857 | -29.002 |
| SRR5919227 | 120.5462 | -28.0505 |
| SRR5919245 | 138.7976 | -30.7757 |
| SRR5919246 | 138.5673 | -31.3277 |
| SRR5919249 | 150.2682 | -33.6528 |
| SRR5919342 | 138.727 | -34.9334 |
| SRR5919346 | 149.5972 | -30.2007 |
| SRR5923059 | 150.7254 | -33.609 |
| SRR5947207 | -123.631 | 39.7392 |
| SRR7777325 | -123.631 | 39.7392 |
| SRR908208 | 34.82 | 31.99 |
| SRR908211 | 34.82 | 31.99 |
| SRR908272 | 34.82 | 31.99 |
| SRR9179559 | 8.518333 | 47.42694 |
| SRR922014 | 9.54406 | 51.7485 |
| DRR101465 | 119.1364 | 32.47979 |
| DRR160706 | 130.766 | 32.889 |
| DRR160713 | 139.539 | 35.735 |
| ERR4661011 | 37.353 | -3.076 |
| SRR10011770 | -105.37 | 39.99 |
| SRR10011774 | -96.57 | 39.09 |
| SRR10011778 | -72.19 | 42.53 |
| SRR10011781 | -71.75 | 43.94 |
| SRR10968227 | 14.70663 | 48.66597 |
| SRR10968229 | 14.70988 | 48.66638 |
| SRR10968252 | 14.70533 | 48.66709 |
| SRR10968254 | 14.70437 | 48.6673 |
| SRR10968258 | 14.70741 | 48.66365 |
| SRR10968259 | 14.70905 | 48.66487 |
| SRR10968263 | 14.70873 | 48.6647 |
| SRR10968265 | 14.70922 | 48.66598 |
| SRR10968266 | 14.70688 | 48.66552 |
| SRR10968267 | 14.70354 | 48.66689 |
| SRR10968268 | 14.7076 | 48.66613 |
| SRR10968269 | 14.70428 | 48.6667 |
| SRR1157614 | -93.4543 | 47.5062 |
| SRR12102599 | 34.74583 | 30.60917 |
| SRR12284403 | 137.1363 | -30.4704 |
| SRR12418378 | 133.7125 | -23.9778 |
| SRR12473534 | 34.91306 | 31.34917 |
| SRR12473535 | 34.91333 | 31.34583 |
| SRR12473537 | 34.91472 | 31.63028 |
| SRR12473538 | 34.94917 | 30.405 |
| SRR12473539 | 34.95056 | 30.40556 |
| SRR12823105 | -119.737 | 46.2528 |
| SRR13062010 | 26.52 | -25.99 |
| SRR2546422 | -118.43 | 34.09 |
| SRR5262250 | -79.8436 | 9.1086 |
| SRR5438270 | -123.631 | 39.7392 |
| SRR5915952 | 147.3106 | -42.3846 |
| SRR5916164 | 145.0819 | -41.6556 |
| SRR5916310 | 147.4015 | -42.2844 |
| SRR5918914 | 150.2678 | -33.653 |
| SRR5919096 | 135.7192 | -14.1952 |
| SRR5919159 | 130.9937 | -31.4092 |
| SRR5919200 | 131.0516 | -25.3504 |
| SRR5919212 | 152.8789 | -27.3861 |
| SRR5919288 | 150.6731 | -35.163 |
| SRR7457710 | 119.8167 | 34.2487 |
| SRR7595584 | 149.5972 | -30.2007 |
| SRR8821151 | 146.8927 | -42.2715 |
| SRR908279 | 34.82 | 31.99 |
| SRR908281 | 34.82 | 31.99 |
| SRR908290 | 34.82 | 31.99 |
| SRR908291 | 34.82 | 31.99 |
| SRR9179560 | 8.518333 | 47.42694 |
| DRR101456 | 119.1364 | 32.47979 |
| DRR101457 | 119.1364 | 32.47979 |
| DRR101458 | 119.1364 | 32.47979 |
| DRR101459 | 119.1364 | 32.47979 |
| DRR101460 | 119.1364 | 32.47979 |
| DRR101462 | 119.1364 | 32.47979 |
| DRR101463 | 119.1364 | 32.47979 |
| DRR101464 | 119.1364 | 32.47979 |
| DRR160704 | 130.766 | 32.889 |
| DRR160705 | 130.766 | 32.889 |
| DRR160707 | 130.766 | 32.889 |
| DRR160708 | 130.766 | 32.889 |
| DRR160709 | 139.539 | 35.735 |
| DRR160710 | 139.539 | 35.735 |
| DRR160711 | 139.539 | 35.735 |
| DRR160712 | 139.539 | 35.735 |
| DRR160714 | 139.539 | 35.735 |
| DRR160716 | 140.244 | 38.249 |
| DRR160717 | 140.244 | 38.249 |
| DRR160718 | 140.244 | 38.249 |
| DRR160719 | 140.244 | 38.249 |
| DRR160720 | 140.244 | 38.249 |
| ERR4660996 | 37.353 | -3.076 |
| ERR4660997 | 37.353 | -3.076 |
| ERR4660998 | 37.353 | -3.076 |
| ERR4660999 | 37.353 | -3.076 |
| ERR4661000 | 37.353 | -3.076 |
| ERR4661001 | 37.353 | -3.076 |
| ERR4661002 | 37.353 | -3.076 |
| ERR4661004 | 37.353 | -3.076 |
| ERR4661006 | 37.353 | -3.076 |
| ERR4661007 | 37.353 | -3.076 |
| ERR4661008 | 37.353 | -3.076 |
| ERR4661009 | 37.353 | -3.076 |
| ERR4661010 | 37.353 | -3.076 |
| ERR4661012 | 37.353 | -3.076 |
| ERR4661013 | 37.353 | -3.076 |
| ERR4661014 | 37.353 | -3.076 |
| ERR4661015 | 37.353 | -3.076 |
| SRR10011775 | -96.57 | 39.09 |
| SRR10011779 | -72.19 | 42.53 |
| SRR10586121 | 103.7 | 1.42 |
| SRR10586123 | 103.7 | 1.42 |
| SRR10586124 | 103.7 | 1.42 |
| SRR10586125 | 103.7 | 1.42 |
| SRR10586126 | 103.7 | 1.42 |
| SRR10586127 | 103.7 | 1.42 |
| SRR10586129 | 103.7 | 1.42 |
| SRR10586130 | 103.7 | 1.42 |
| SRR10586131 | 103.7 | 1.42 |
| SRR10586135 | 103.7 | 1.42 |
| SRR10586136 | 103.7 | 1.42 |
| SRR11271807 | -71.68 | -38.82 |
| SRR1152189 | -89.944 | 40.296 |
| SRR1152189 | -89.944 | 40.296 |
| SRR1157615 | -93.45 | 47.51 |
| SRR1157617 | -93.489 | 47.50505 |
| SRR12102601 | 34.91333 | 31.34583 |
| SRR12284400 | 137.1363 | -30.4704 |
| SRR12284401 | 137.1363 | -30.4704 |
| SRR12284402 | 137.1363 | -30.4704 |
| SRR12284404 | 137.1363 | -30.4704 |
| SRR12284409 | 137.1363 | -30.4704 |
| SRR12284410 | 137.1363 | -30.4704 |
| SRR12284412 | 137.1363 | -30.4704 |
| SRR12418371 | 145.3131 | -37.9265 |
| SRR12418372 | 145.3131 | -37.9265 |
| SRR12418374 | 144.4701 | -37.4464 |
| SRR12418376 | 144.4701 | -37.4464 |
| SRR12418384 | 145.3131 | -37.9265 |
| SRR12418385 | 133.7125 | -23.9778 |
| SRR12418386 | 133.7125 | -23.9778 |
| SRR12473530 | 34.97481 | 29.94152 |
| SRR12473531 | 34.76735 | 30.78534 |
| SRR12473532 | 34.74583 | 30.60917 |
| SRR12473533 | 34.74583 | 30.60917 |
| SRR12473536 | 34.75278 | 31.25333 |
| SRR12473540 | 34.93304 | 31.64222 |
| SRR12473541 | 34.93501 | 31.64237 |
| SRR12823096 | -119.737 | 46.2528 |
| SRR12823097 | -119.737 | 46.2528 |
| SRR12823098 | -119.737 | 46.2528 |
| SRR12823099 | -119.737 | 46.2528 |
| SRR12823100 | -119.737 | 46.2528 |
| SRR12823101 | -119.737 | 46.2528 |
| SRR12823102 | -119.737 | 46.2528 |
| SRR12823104 | -119.737 | 46.2528 |
| SRR12823106 | -119.737 | 46.2528 |
| SRR12823107 | -119.737 | 46.2528 |
| SRR12823108 | -119.737 | 46.2528 |
| SRR12823109 | -119.737 | 46.2528 |
| SRR12823111 | -119.737 | 46.2528 |
| SRR12823112 | -119.737 | 46.2528 |
| SRR12823113 | -119.737 | 46.2528 |
| SRR12823114 | -119.737 | 46.2528 |
| SRR12823115 | -119.737 | 46.2528 |
| SRR12823117 | -119.737 | 46.2528 |
| SRR12823118 | -119.737 | 46.2528 |
| SRR12823119 | -119.737 | 46.2528 |
| SRR13062007 | 26.52 | -25.99 |
| SRR13062009 | 26.52 | -25.99 |
| SRR2546421 | -118.43 | 34.09 |
| SRR5204863 | -123.631 | 39.7392 |
| SRR5205085 | -123.631 | 39.7392 |
| SRR5205807 | -123.631 | 39.7392 |
| SRR5205809 | -123.631 | 39.7392 |
| SRR5205814 | -123.631 | 39.7392 |
| SRR5205823 | -123.631 | 39.7392 |
| SRR5207237 | -123.631 | 39.7392 |
| SRR5207244 | -123.631 | 39.7392 |
| SRR5207245 | -123.631 | 39.7392 |
| SRR5207247 | -123.631 | 39.7392 |
| SRR5207248 | -123.631 | 39.7392 |
| SRR5207249 | -123.631 | 39.7392 |
| SRR5207253 | -123.631 | 39.7392 |
| SRR5262244 | -79.8436 | 9.1086 |
| SRR5262247 | -79.8436 | 9.1086 |
| SRR5436189 | -123.631 | 39.7392 |
| SRR5436305 | -123.631 | 39.7392 |
| SRR5436309 | -123.631 | 39.7392 |
| SRR5436853 | -123.631 | 39.7392 |
| SRR5437016 | -123.631 | 39.7392 |
| SRR5437588 | -123.631 | 39.7392 |
| SRR5437660 | -123.631 | 39.7392 |
| SRR5437667 | -123.631 | 39.7392 |
| SRR5437762 | -123.631 | 39.7392 |
| SRR5437763 | -123.631 | 39.7392 |
| SRR5437764 | -123.631 | 39.7392 |
| SRR5437773 | -123.631 | 39.7392 |
| SRR5437868 | -123.631 | 39.7392 |
| SRR5437870 | -123.631 | 39.7392 |
| SRR5437899 | -123.631 | 39.7392 |
| SRR5438045 | -123.631 | 39.7392 |
| SRR5438046 | -123.631 | 39.7392 |
| SRR5438067 | -123.631 | 39.7392 |
| SRR5438093 | -123.631 | 39.7392 |
| SRR5438094 | -123.631 | 39.7392 |
| SRR5438105 | -123.631 | 39.7392 |
| SRR5438128 | -123.631 | 39.7392 |
| SRR5438132 | -123.631 | 39.7392 |
| SRR5438309 | -123.631 | 39.7392 |
| SRR5438445 | -123.631 | 39.7392 |
| SRR5438477 | -123.631 | 39.7392 |
| SRR5438552 | -123.631 | 39.7392 |
| SRR5438874 | -123.631 | 39.7392 |
| SRR5438876 | -123.631 | 39.7392 |
| SRR5919112 | 141.9763 | -35.1164 |
| SRR5919199 | 131.0516 | -25.3504 |
| SRR5919201 | 131.0423 | -25.351 |
| SRR5919202 | 131.0516 | -25.3504 |
| SRR5947208 | -123.631 | 39.7392 |
| SRR5947209 | -123.631 | 39.7392 |
| SRR7042337 | -123.63 | 39.74 |
| SRR7042339 | -123.63 | 39.74 |
| SRR7042340 | -123.63 | 39.74 |
| SRR7042342 | -123.63 | 39.74 |
| SRR7042345 | -123.63 | 39.74 |
| SRR7042346 | -123.63 | 39.74 |
| SRR7042350 | -123.63 | 39.74 |
| SRR7042353 | -123.63 | 39.74 |
| SRR7042359 | -123.63 | 39.74 |
| SRR7042360 | -123.63 | 39.74 |
| SRR7042362 | -123.63 | 39.74 |
| SRR7042364 | -123.63 | 39.74 |
| SRR7042367 | -123.63 | 39.74 |
| SRR7042372 | -123.63 | 39.74 |
| SRR7042376 | -123.63 | 39.74 |
| SRR7042377 | -123.63 | 39.74 |
| SRR7042382 | -123.63 | 39.74 |
| SRR7042387 | -123.63 | 39.74 |
| SRR7042390 | -123.63 | 39.74 |
| SRR7042393 | -123.63 | 39.74 |
| SRR7448200 | 119.8167 | 34.2487 |
| SRR7448260 | 119.8167 | 34.2487 |
| SRR7448267 | 119.8167 | 34.2487 |
| SRR7448268 | 119.8167 | 34.2487 |
| SRR7448274 | 119.8167 | 34.2487 |
| SRR7448275 | 119.8167 | 34.2487 |
| SRR7448276 | 119.8167 | 34.2487 |
| SRR7448277 | 119.8167 | 34.2487 |
| SRR7448278 | 119.8167 | 34.2487 |
| SRR7457704 | 119.8167 | 34.2487 |
| SRR7457705 | 119.8167 | 34.2487 |
| SRR7457706 | 119.8167 | 34.2487 |
| SRR7457707 | 119.8167 | 34.2487 |
| SRR7457708 | 119.8167 | 34.2487 |
| SRR7457709 | 119.8167 | 34.2487 |
| SRR7457711 | 119.8167 | 34.2487 |
| SRR7457712 | 119.8167 | 34.2487 |
| SRR7457713 | 119.8167 | 34.2487 |
| SRR7595568 | 146.8927 | -42.2715 |
| SRR8821132 | 149.5972 | -30.2007 |
| SRR8821142 | 147.3189 | -41.7775 |
| SRR908273 | 34.82 | 31.99 |
| SRR908275 | 34.82 | 31.99 |
| SRR908276 | 34.82 | 31.99 |
| SRR9179561 | 8.518333 | 47.42694 |
| SRR9179562 | 8.518333 | 47.42694 |
| SRR988079 | -97.519 | 34.97567 |
